# A landmark-free morphometrics pipeline for high-resolution phenotyping: application to a mouse model of Down syndrome

**DOI:** 10.1242/dev.188631

**Published:** 2021-03-12

**Authors:** Nicolas Toussaint, Yushi Redhead, Marta Vidal-García, Lucas Lo Vercio, Wei Liu, Elizabeth M. C. Fisher, Benedikt Hallgrímsson, Victor L. J. Tybulewicz, Julia A. Schnabel, Jeremy B. A. Green

**Affiliations:** 1 School of Biomedical Engineering and Imaging Sciences, King's College London, UK; 2 Centre for Craniofacial Biology & Regeneration, King's College London, UK; 3 The Francis Crick Institute, London NW1 1AT, UK; 4 Department of Cell Biology & Anatomy, University of Calgary, Calgary AB T2N 4N1, Canada; 5 Department of Neurodegenerative Disease, Institute of Neurology, University College London, London WC1N 3BG, UK; 6 Department of Immunology & Inflammation, Imperial College London, London W12 0NN, UK

**Keywords:** Craniofacial, Cranium, Down syndrome, Morphometrics, Mouse model, Phenotyping

## Abstract

Characterising phenotypes often requires quantification of anatomical shape. Quantitative shape comparison (morphometrics) traditionally uses manually located landmarks and is limited by landmark number and operator accuracy. Here, we apply a landmark-free method to characterise the craniofacial skeletal phenotype of the Dp1Tyb mouse model of Down syndrome and a population of the Diversity Outbred (DO) mouse model, comparing it with a landmark-based approach. We identified cranial dysmorphologies in Dp1Tyb mice, especially smaller size and brachycephaly (front-back shortening), homologous to the human phenotype. Shape variation in the DO mice was partly attributable to allometry (size-dependent shape variation) and sexual dimorphism. The landmark-free method performed as well as, or better than, the landmark-based method but was less labour-intensive, required less user training and, uniquely, enabled fine mapping of local differences as planar expansion or shrinkage. Its higher resolution pinpointed reductions in interior mid-snout structures and occipital bones in both the models that were not otherwise apparent. We propose that this landmark-free pipeline could make morphometrics widely accessible beyond its traditional niches in zoology and palaeontology, especially in characterising developmental mutant phenotypes.

## Abstract (precis)

**Summary:** This paper presents a new computational pipeline for comparing and mapping global and local anatomical shape differences that is higher-resolution and requires less expertise and labour than traditional landmarking methods.

## INTRODUCTION

Morphometrics, the quantitative comparison of biological shapes, is well established in the fields of palaeontology and evolutionary biology to quantify and understand morphological phenotypes (Cooke and Terhune, 2015). Landmark positions are recorded on digital two-dimensional (2D) or three-dimensional (3D) images (obtained by photography, X-ray or MRI methods) and their spatial distributions are then analysed through Euclidean distance matrix analysis (EDMA) or Procrustes Superimposition (PS) (Webster and Sheets, 2010). Morphometrics is less used in other fields, such as genetics and developmental biology. This may be because current morphometric methodologies, although powerful, have several limitations.

First, the number of landmarks always reflects a compromise between precision, which needs many anatomical landmarks to be located, and ease-of-use, which limits those numbers. Few landmarks result in large gaps between them, making mapping not only imprecise but typically so dominated by overall scale (size) effects that these must be removed by scale normalisation before further analysis can be performed. However, separating global scale from other shape differences is a purely artificial measure that may or may not reflect the mechanism of difference, as it is not possible to fully disentangle global from regional differences. Having many landmarks is therefore preferable because differences are then mapped where, or close to where, they actually occur. This improves not only spatial resolution and the fidelity of shape-difference visualisation, but also has the potential to avoid the need to separate size and shape change as all mapping can be highly local. However, with landmark-based methods, this is highly laborious or even unfeasible. Typically, some tens of landmarks are located manually, which takes considerable anatomical knowledge and training and time.

Second, an anatomical landmark may be absent from an individual owing to natural variation, engineered mutation or pathology. Third, landmarks can be sparse in anatomical structures where they are hard to define: smooth surfaces do not have easily defined landmarks. Sparseness is a particular problem in soft tissues and embryos, with numerous featureless curved surfaces. Semi-landmarks interpolated between landmarks (Andresen et al., 2000; Bookstein, 1997; Frangi et al., 2003) reduce this problem but still leave gaps (Palci and Lee, 2019). Fourth, manual landmark-based methods are inevitably susceptible to both inter- and intra-operator variability, which can be as big as the biological variability between subjects (Percival et al., 2014; Shearer et al., 2017; von Cramon-Taubadel et al., 2007). Together, these limitations suggest that there is a need for automated, ideally landmark-free, high-resolution methods. Landmark-free methods have been developed by the neuroimaging community to quantify the size and shape of the brain precisely because its relatively smooth shape hampers the definition of reliable landmarks (Bron et al., 2015; Routier et al., 2014), but these methods have yet to be applied more widely and have not been directly compared with the landmark-based approach.

One of the most common human dysmorphologies is the craniofacial phenotype associated with Down syndrome (DS). Individuals with DS, currently ∼1 in 800 births (Antonarakis, 2017), have characteristic features – flattened midface with low nose bridge, front-to-back shortened skull (brachycephaly) and slightly hooded eyelids (Korenberg et al., 1994). Although the craniofacial features affect everyone with DS, this phenotype is not well understood either genetically or developmentally. DS is caused by trisomy of human chromosome 21 (Hsa21) which carries 232 protein-coding genes (Ensembl genome assembly GRCh38; Antonarakis, 2017; Lejeune et al., 1959). It is thought that the presence of a third copy of one or more of these genes gives rise to the individual defects observed in DS, but the crucial dosage-sensitive genes are not known (Lana-Elola et al., 2016, 2011; Watson-Scales et al., 2018).

Analysis of the Ts65Dn mouse strain, an early mouse model of DS, using landmark-based morphometrics showed craniofacial dysmorphology, which was ascribed to defects in neural crest migration (Hill et al., 2007, 2009; Richtsmeier et al., 2000; Roper et al., 2009). This strain has an extra copy of 132 Hsa21-orthologous protein-coding genes on mouse chromosome 16 (Mmu16), thereby mimicking part of the increased gene dosage in DS. However, these mice also have a third copy of 46 protein-coding genes on Mmu17 that are not orthologous to Hsa21 (Duchon et al., 2011; Reinholdt et al., 2011), thus it is unclear whether the phenotypic changes seen in Ts65Dn mice are due to increased dosage of Hsa21-orthologous genes. More recently, we and others have generated improved mouse models of DS by using precise chromosome engineering techniques to make mouse strains with an extra copy of each of the three Hsa21-orthologous regions of the mouse genome on Mmu10, Mmu16 and Mmu17 (Herault et al., 2017; Lana-Elola et al., 2016; Li et al., 2007; Yu et al., 2010). Dp(16)1Yey and Dp1Tyb mice each have an extra copy of the largest of these, the entire Hsa21-orthologous region of Mmu16, containing 147 protein-coding genes (Lana-Elola et al., 2016; Li et al., 2007). Landmark-based morphometric analysis of Dp(16)1Yey mice showed craniofacial dysmorphology which resembled the DS phenotype (Starbuck et al., 2014). This dysmorphology in Dp(16)1Yey mice was statistically significant (with multiple linear distances between landmarks differing from wild type in all regions measured) yet quantitatively subtle, with an average landmark-to-landmark distance difference of only 7% between mutant and wild-type (WT) control mice (Starbuck et al., 2014).

In this paper, we describe a convenient pipeline we have developed for landmark-free morphometric analysis based on an approach used for brain imaging (Durrleman et al., 2014). We compare our method with the traditional landmark-based morphometric approach, focusing initially on the characterisation of the craniofacial phenotype of the Dp1Tyb mouse model of DS, which is genetically almost identical to Dp(16)1Yey mice, but has not been previously analysed (Lana-Elola et al., 2016). To evaluate the landmark-free approach in a larger sample, we quantified subtle patterns of shape variation in a relatively larger sample of Diversity Outbred (DO) mice. DO mice are derived from the same eight founder strains as the Collaborative Cross (CC) inbred strains (Churchill et al., 2012), which included three mouse subspecies, resulting in a population with relatively high genetic and morphological diversity that resembles the diversity found in natural populations (Churchill et al., 2012; Katz et al., 2020).

We find that the landmark-free analysis gives separation by shape both between Dp1Tyb and WT mice, and in allometry (size-dependent shape variation) and sexual dimorphism in the skulls of DO mice. Our results show that landmark-free analysis reveals differences at least as clearly as those seen by landmark-based analysis, while delivering a number of operational advantages. We demonstrate a new tool (‘local stretch’ mapping) that avoids the need to separate scale changes from shape changes, provides a high fidelity and intuitive visualisation tool and localises abnormalities in the DS model to cranial vault expansion and mid-face and occipital contraction.

## RESULTS

### Landmark-based and landmark-free analysis of Dp1Tyb skulls

To phenotype the Dp1Tyb DS model skulls, we used micro-computed tomography (µCT) to acquire images of the skulls of 16-week old WT and Dp1Tyb mice. We carried out landmark-based analysis in the conventional way (Kristensen et al., 2008), marking the location of 68 landmarks on the cranium and 17 on the mandible (Fig. S1). Crania and mandibles were analysed separately as their relative position varied from subject to subject. Landmarks for all crania and mandibles were aligned using PS, and these data were used for further statistical analysis of size and shape.

**Fig. 1.**
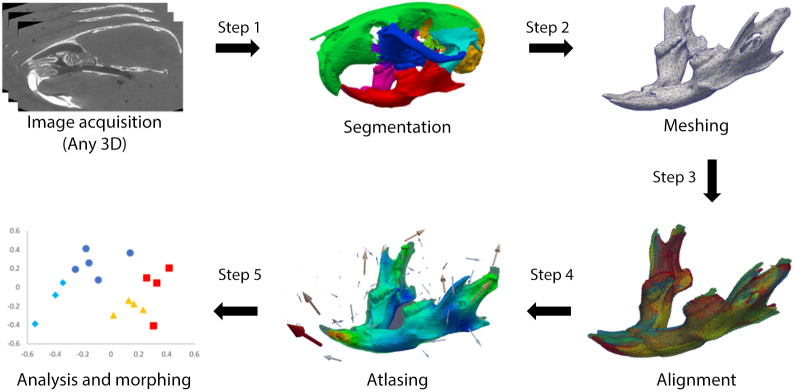
**Stages of processing in the landmark-free approach.** Step 1, extraction of region of interest. Initial thresholding of µCT image was used to make a binary mask and regions of the mask were separated by bone density using secondary thresholding, with some manual clean-up based on known anatomy. A region of interest (in this example the mandible in red) was chosen for further analysis. Step 2, mesh generated and decimated by a factor of 0.0125 to reduce data file size. Step 3, meshes of all subjects aligned either using rigid body alignment with no scaling or similarity alignment with scaling. Step 4, atlas construction (arrows represent momenta). Step 5, statistical analysis and visualisation of shape data.

For the landmark-free approach we developed a pipeline based on previous approaches in morphometrics and neuroimaging (outlined in Fig. 1 and Table 1). For details, the reader is directed to the relevant section of Materials and Methods and to the supplementary Materials and Methods but, in brief, following thresholding to extract the skull structures from the µCT images, cartilaginous structures were removed (Fig. S2) and the images segmented using bone density to separate the mandibles from the crania (Fig. 1, step 1). Triangulated meshes were generated from the surfaces (including internal surfaces) of the cranium for all subjects, and the mandibles for the WT and Dp1Tyb specimens decimated and cleaned (Fig. 1, step 2), aligned (and scaled where appropriate – see below) (Fig. 1, step 3). The meshes were used for the construction of an atlas (mean shape) for the crania and mandibles of the WT and Dp1Tyb skulls (Fig. 1, step 4). Atlas construction was based on the Deformetrica algorithm (Durrleman et al., 2014) which works by defining a flow field (tensor) that conforms to its shape and quantifies deformations from it to each subject recorded as momentum vectors (momenta – see Materials and Methods and Supplementary Materials and Methods). Note that this flow field fills the entire space of the mesh and its surroundings and is thus more similar to the deformation grids of D'Arcy Thompson (https://medium.com/miccai-educational-initiative/a-beginners-guide-to-shape-analysis-using-deformetrica-fa9e346357b7; see also Discussion; Thompson, 1942) than to landmark-based methods. The initial output from this atlas consisted of the average mesh for the whole population (based on averaging the tensors), a set of control points corresponding to areas with the greatest variability between subjects, and momenta for each control point describing the directional variation of the shape from the average. The average mesh, the control points and the momenta were used for further statistical analysis, with the momenta applied to deform the population average mesh to generate average meshes for each of WT and Dp1Tyb groups preserving one-to-one correspondence of mesh vertices. We performed principal component analysis (PCA) and used a multiple permutations test on a stratified k-fold cross validation classifier to test for significance (Fig. 1, step 5). To control for overfitting (a risk when the number of measurements substantially exceeds the number of subjects), we compared the PCA difference vector magnitude between the two genotype groups with that of 1000 randomly scrambled groups. We found that the distribution was normal and that the genotype difference vector was more than 3.5 standard deviations away from the mean vector of the 1000 scrambled groups for both cranium and mandible, thus showing that overfitting is unlikely to be a significant factor (Fig. S3).

**Table 1.**
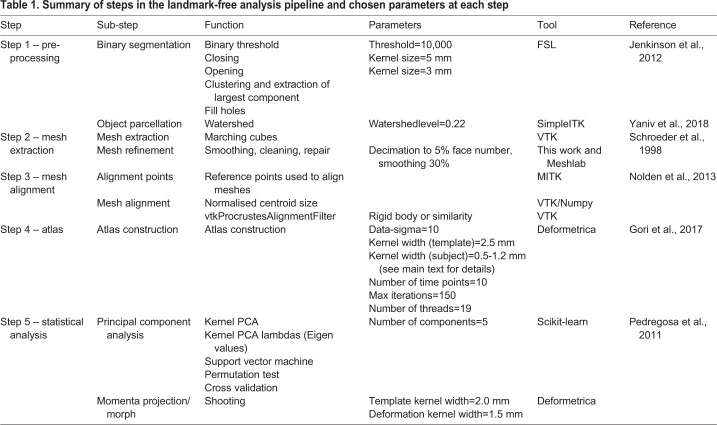
**Summary of steps in the landmark-free analysis pipeline and chosen parameters at each step**

### Size differences: Dp1Tyb mice have significantly smaller crania and mandibles

**Fig. 2.**
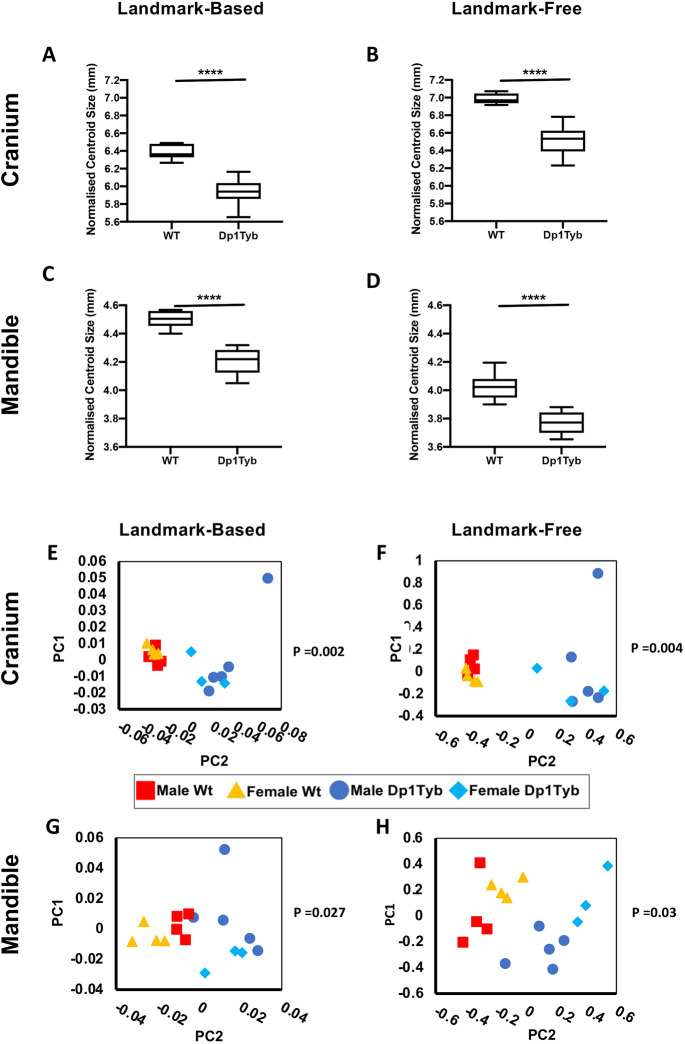
**Decreased size and altered shape of Dp1Tyb crania and mandibles.** (A-D) Normalised centroid sizes of WT and Dp1Tyb crania (A,B) and mandibles (C,D) determined using landmark-based (A,C) and landmark-free (B,D) methods. Data shown as box and whiskers plots indicating the 25% and 75% centiles (box), range of all data points (whiskers) and the median (black line). Statistical significance was calculated using a two-tailed unpaired *t*-test; ****P<0.0001. (E-H) PCA (first two components) of Procrustes-aligned shapes of WT and Dp1Tyb crania (E,F) and mandibles (G,H) determined using landmark-based (E,G) and landmark-free (F,H) methods. Statistical significance (*P*-values) of WT versus Dp1pTyb differences was calculated using a permutation test. Sample size: *n*=8 of each genotype.

Comparison of object sizes in morphometric analysis is usually carried out using centroid size (the square-root of the sum of the squares of landmark distances to the subject centroid) (Klingenberg, 2016). However, as centroid sizes scale with the number of landmarks, sizes cannot be compared when different numbers of landmarks are used; here, in the landmark-based method we used 68 and 17 landmarks for the cranium and mandible, respectively, whereas in the landmark-free method we used ∼19,000 and ∼16,000 mesh vertices for the same two structures. To generate somewhat more comparable measures, we divided the centroid sizes by the number of landmarks or mesh vertices, respectively, to derive ‘normalized’ centroid sizes, and used these (separately, as they do not provide strict comparability between methods) to compare Dp1Tyb and sibling control specimens. Landmark-based normalized centroid size comparison showed that the crania and mandibles of Dp1Tyb mice were both significantly smaller than those of WT mice (Fig. 2A,C), recapitulating the overall reduction in skull size found in humans with DS and as well as in other models of DS (Hill et al., 2007; Richtsmeier et al., 2000, 2002; Starbuck et al., 2014; Suri et al., 2010). The landmark-free analysis likewise showed that Dp1Tyb crania and mandibles were significantly smaller than WT (Fig. 2B,D; Table S1). The normalised centroid sizes between the landmark-based and landmark-free methods were different. This is unsurprising given the many extra measurements used in the landmark-free method. However, they both showed the same magnitude of differences between genotypes of ∼7% (Table S1).

### Shape differences: Dp1Tyb mice have altered crania and mandibles

Both the size difference and gross shape differences were clearly visualised by animated morphing between the mean shapes of WT and Dp1Tyb specimens (generated in the landmark-free pipeline) for the cranium and the mandible (Movies 1 and 2). The overall decrease in size going from WT to Dp1Tyb crania or mandibles was readily apparent and some shape changes could also be seen, although the latter were more subtle.

To quantify shape differences statistically, shape was separated from size by scaling the data to equalise centroid sizes (Procrustes alignment). To analyse residual shape differences between genotypes, we used PCA. Both methods and both genotypes showed no separation by sex in the cranium and only a subtle separation in the mandible. Moreover, considering each sex separately, genotype-dependent separations were similar, showing that they were not sex-dependent. The sexes were therefore pooled for statistical analysis. Both landmark-based and landmark-free methods showed statistically significant differences in shape between Dp1Tyb and WT mice in both crania and mandibles (Fig. 2E-H). Plots of the first two principal components identified by the two different methods looked similar, with tighter clustering of specimens for cranium than for mandible.

### Shape difference localisation: Dp1Tyb mice recapitulate aspects of human DS craniofacial dysmorphology

**Fig. 3.**
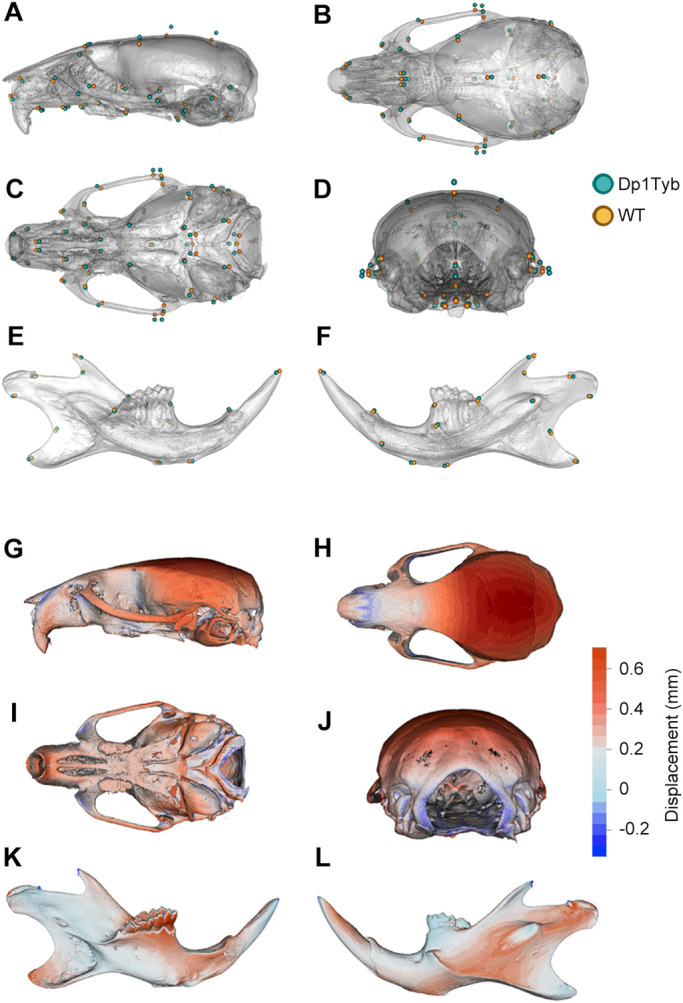
**Visualisation of altered shape of Dp1Tyb crania and mandibles determined using landmark-based analysis.** (A-F) Mean landmark configurations of WT (orange) and Dp1Tyb (cyan) crania (A-D) and mandibles (E,F), showing lateral (A), superior (B), inferior (C) and rear (D) views of the cranium, and lingual (E) and buccal (F) views of the mandible. (G-L) Displacement heatmaps after global size differences have been regressed out, produced by superimposing the mean shapes of the WT and Dp1Tyb crania (G-J) and mandibles (K,L), showing lateral (G), superior (H), inferior (I) and rear (J) views of the cranium, and lingual (K) and buccal (L) views of the mandible. Red and blue represent the distribution of expansion and contraction, respectively, in Procrustes (shape) distance. Regions of the Dp1Tyb mesh outside the WT mesh are coloured red, whereas any parts inside the WT mesh are coloured blue, thereby showing displacement relative to WT.

To characterise the shape differences anatomically, we first overlaid the mean landmark configurations from Dp1Tyb and WT crania and mandibles (Fig. 3A-F). Second, we applied an established thin-plate spline interpolation and comparison package (Morpho R – see Materials and Methods) to the landmark data to generate displacement heatmaps (Fig. 3G-L). Direct inspection revealed that the morphological differences between Dp1Tyb and WT skulls were broadly distributed and relatively subtle, consistent both with previously reported mouse DS models and with the human phenotype (Fischer-Brandies, 1988; Fischer-Brandies et al., 1986; Suri et al., 2010). The maps revealed that Dp1Tyb mice have a more domed neurocranium (cyan points at the top-right of Fig. 3A, dark red regions in Fig. 3G,H,J). The cranial doming in combination with the overall smaller size compared with WT mice constitute a net anteroposterior shortening in Dp1Tyb mice, i.e. brachycephaly, a predominant feature of the human DS phenotype. This method also indicated an almost unchanged cranial base (Fig. 3I) and some contraction (anterior movement) concentrated around the magnum foramen in the occipital bone of Dp1Tyb crania (points at right of Fig. 3C, blue colour on the right of Fig. 3I and in Fig. 3J). These maps also showed a smaller snout in Dp1Tyb mice as a result of a reduction in size of the nasal bones (Fig. 3G,H). Although not evident in the heatmaps, the Dp1Tyb cranium was wider, as can be seen by the displacement of the zygomatic process landmarks laterally (Fig. 3C,D). The reduced snout and facial widening in combination with the overall smaller size of the Dp1Tyb crania mimics the ‘mid-face hypoplasia’ of human DS. The Dp1Tyb mandibles had a small shape change in the alveolar ramus region and the condylar process, but these changes were all extremely subtle (Fig. 3E,F,K,L).

**Fig. 4.**
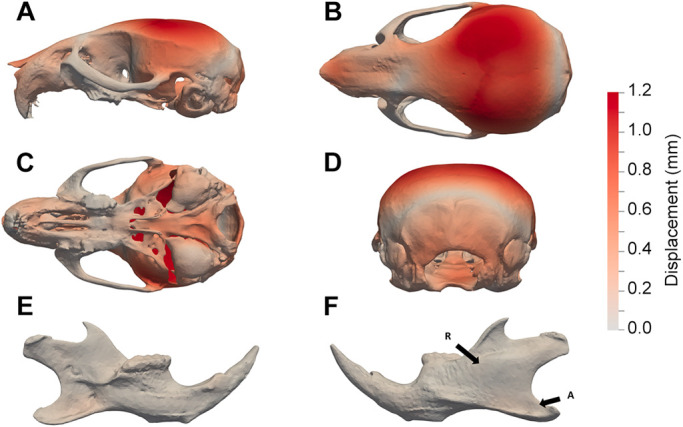
**Visualisation of altered shape of Dp1Tyb crania and mandibles determined using landmark-free analysis.** (A-F) Displacement heatmaps after global size differences have been regressed out estimated using output momenta from the current-based atlas construction, showing locations of shape differences between WT and Dp1Tyb crania (A-D) and mandibles (E,F), showing lateral (A), superior (B), inferior (C) and rear (D) views of the cranium, and lingual (E) and buccal (F) views of the mandible. A-F are on the same colour scale, which indicates the magnitude of the shape differences as displacement. Arrows R and A indicate the ramus and angular process, respectively.

Next, we made heatmaps based on the higher-resolution landmark-free method. Displacement maps together with the morphing movies visualised the distances between the two mean meshes. Deformetrica outputs net displacement rather than movement towards or away from the shape centroid so that only one colour appears in these maps, but the direction of difference is clearly shown in Movies 1-5. The landmark-free analysis showed changes mostly similar to those found using the landmark-based method including the same relative doming of the neurocranium and shortening of the nasal and maxillary processes (Fig. 4A-D; Movie 3).

Differences between Dp1Tyb and WT mandibles were overall much more subtle (Fig. 4E,F; Movie 4), consisting of a few tens of microns only. Generating a video in which the shape change of the mandible was exaggerated by a factor of three helped localise shape changes (Movie 5). Thus, we see the expansion buccally (cheek-wards) of the inferior portion of the ramus (the region immediately posterior to the molars), the contraction of the angular process, lingual movement of the molar ridge and widening of the incisor alveolus. The expansion of the ramus had not shown up in the landmark-based method because there were no landmarks in this region.

### New shape change information: the landmark-free method maps in-plane deformation

**Fig. 5.**
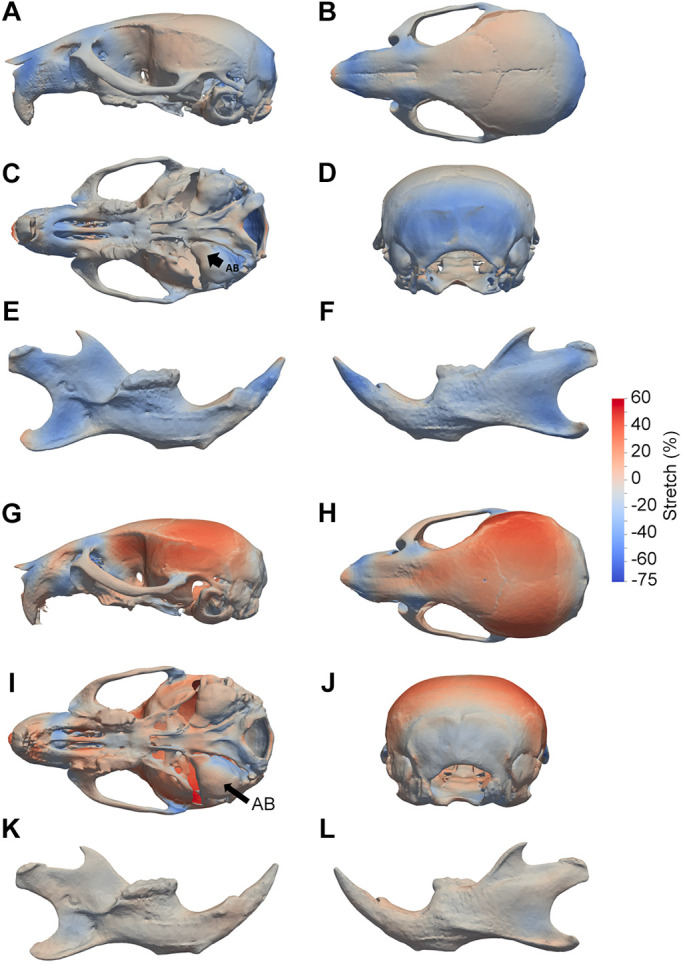
**Altered surface stretch in Dp1Tyb crania and mandibles determined using landmark-free analysis.** (A-L) Heatmaps show unscaled (A-F) or scaled (G-L) (i.e. with global size difference regressed out) surface stretch between WT and DS crania (A-D,G-J) and mandibles (E,F,K,L), showing lateral (A,G), superior (B,H), inferior (C,I) and rear (D,J) views of the cranium, and lingual (E,K) and buccal (F,L) views of the mandible. Stretch changes were estimated using output momenta from the current-based atlas construction. Arrow AB indicates the auditory bulla.

One of the reasons for separating shape difference from size difference in morphometrics is that a simple uniform scale change would appear, artefactually, as change localised distal to whatever point was used as the common frame of reference (i.e. if the centroids are used, there is an increasing centre-to-edge gradient – see demonstration in Fig. S4). Scaling avoids this problem but throws away the ‘ground truth’ of the differences. One solution is to find a way of showing size changes entirely locally, capturing surface ‘stretch’ as a measure of local growth differences (either by cell proliferation or by other mechanisms) between specimens. This is also likely to reflect real biological differences which arise in development due to different localised growth. This is not possible with landmarks but can be done within the high-resolution landmark-free method where a high density of control points is used to guide an even higher density of mesh vertices. Thus, we calculated and mapped local differences in mesh vertex spacing. We used the spacing to generate a heatmap without the need for scaling. The results are shown in Fig. 5A-F and Movies 1, 2 and 6 (see also Movie 7 for another way of displaying the data). These maps clearly show that the phenotype is almost entirely a size or growth deficit (which may or may not be a cell proliferation deficit) in three main regions: the occipital region posterior to the auditory bulla, the facial bones and hard palate. The auditory bulla itself is smaller in Dp1Tyb than in wild type, but approximately in proportion to the overall smaller skull size; whether this bears any relation to the otitis media often seen in DS individuals remains unclear. Expansion in the mid-cranial vault is minimal. This representation can be compared with the heatmaps generated on size-scaled data (Fig. 5G-L and Movies 8-10): in these the colour emphasises the expansion of the cranial vault, but this is a relative rather than absolute expansion and thus does not correspond to actual growth.

### Landmark-free shape quantification compared with manual and automated landmarking on a larger dataset

Although the above analysis demonstrated the applicability of the landmark-free pipeline on what would be a typical sample size for a mutant study, more thorough validation of our novel landmark-free pipeline for morphometric analysis required determination that the method captures shape variation that is comparable with the gold standard, geometric morphometric analysis of 3D landmark data (Mitteroecker and Gunz, 2009), on a much larger dataset. To this end, we quantified shape variation that co-varies with size (allometry) and sexual dimorphism in a larger sample (*n*=95) of DO mice (see Materials and Methods). Capturing this type of continuous biological variation provides a more powerful test of the sensitivity of a morphometric method than a simple two-population comparison. This was a more challenging task than quantifying the substantial differences between Dp1Tyb and WT skulls, but the larger sample size of DO mice allowed for repeatable estimates of variance components.

**Fig. 6.**
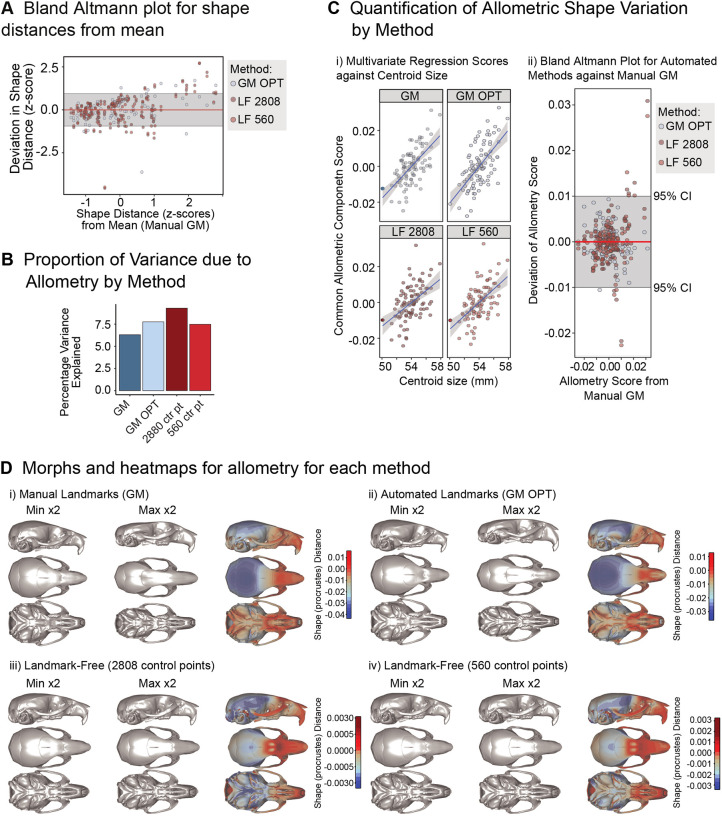
**Validation of landmark-free morphometry in a sample of DO mice.** (A) Bland-Altman plot for shape distances from the mean. Here, the deviations of each method from the manual landmarking-based estimated is plotted against the manual landmarking estimate. Grey region indicates 95% confidence limit for identity. (B) Variance components for allometric shape variation as estimated using the four methods. (C) Common allometric component (CAC) scores for each method plotted against centroid size (grey areas are 95% confidence limits for the slope; i) and a Bland-Altman plot for the CAC score showing agreement among methods (ii). (D) 3D morphs and heatmaps showing the shape variation associated with allometry as estimated by each method: (i) GM=manual landmark-based geometric morphometics; (ii) GP OPT=automated landmark-based geometric morphometrics; (iii) LF 2808 and (iv) LF 560=landmark-free method with 2808 or 560 control points, respectively. ‘Min ×2’ and ‘Max ×2’ label the deformations corresponding to the lower and higher ends of the heatmap colour scales, exaggerated two-fold to more easily visualise the shape differences.

µCT images of the 98 DO mouse skulls were analysed in the landmark-free pipeline described earlier, generating an atlas for this set of specimens. The results for overall shape variation and for allometry are shown in Fig. 6 and those for sexual dimorphism are shown in Fig. S5. We compared shape variation quantifications using three methods: (1) manually obtained 3D landmarks (geometric morphometric; GM); (2) optimized automated landmarks obtained using volumetric registration as recently reported (GM OPT) (Devine et al., 2020); (3) landmark-free analyses using the mesh-based registration introduced in this study, performed using two different densities of control-point grids – the first, at 2808, was similar to the density used for the Dp1Tyb to WT comparison (LF 2808) whereas the second, at 560, was substantially lower (LF 560).

Overall shape variation correlated significantly across methods. However, the correlations were higher between the two sparse landmark sets (GM and GM OPT, *r*=0.75) and between the two landmark-free datasets (LF 2808 and LF 560, *r*=0.94) than between sparse and landmark-free analyses (GM or GM OPT versus LF 2808 or LF 560, *r*=0.54-0.67). This is not surprising, as the greater density of control points means that the landmark-free method is capturing variation that was not present in the sparse landmark set. A Bland-Altman graph (Fig. 6A; Fig. S5), which plots for each specimen the difference between the manual and non-manual shape estimates (in standard deviations) versus the difference between that specimen's deviation from the manual mean (in the same units) shows that the two landmark-free methods approximate the manual landmark-based distances to a similar degree as the automated sparse landmark-based distances: the vast majority of the points are within the 95% confidence limits for identity. In all cases, however, those deviations were larger for specimens at the extremes of the phenotypic range. This is a phenomenon commonly observed in automated landmarking, where extreme measurements tend to be pulled towards the atlas (Bannister et al., 2020; Devine et al., 2020; Li et al., 2017; Percival et al., 2019).

The four methods were similar on estimates of how large a proportion of the total shape variance is attributable to allometry or sexual dimorphism (between 6.5% and 8.1%), although the landmark-free values were somewhat higher (Fig. 6B). This is important because the ability to capture biological variation is as close as one can get to ground truth in such comparisons. The most likely explanations for our finding that the 2808-control point quantification of allometry is 35% higher than that for manual landmarks are that either the manual landmark data includes more noise (measurement error) or that the landmark-free method is capturing allometric variation that is missed in the sparse landmark set. Both are likely true, as suggested by the intermediate value obtained from automated sparse landmarking.

The common allometric component (CAC) score is a commonly used summary measure of multivariate allometric variation (Klingenberg, 2016). CAC values correlate strongly between manual landmarking and the two landmark-free quantifications (*r*=0.83, 0.85) and between the two landmark-free quantifications (*r*=0.98) (Fig. 6C). The Bland-Altman plot for these scores shows good agreement across methods with a few outliers – three individuals in which the landmark-free method produces estimates that depart significantly from those obtained with sparse landmarks. Visualization of the shape variation associated with allometry (Fig. 6D) shows a qualitatively similar morphological pattern with more detail apparent in for the landmark-free method. Quantitative comparison of shape vectors is not possible for these analyses. Similar results are obtained for sexual dimorphism, although this variance component is smaller and more subtle than allometry (Fig. S5).

## DISCUSSION

In this paper, we have presented an adaptation and incorporation of a tensor field-based landmark-free shape comparison methodology (Durrleman et al., 2014) into a pipeline that can compare shapes and provide statistical and other data analyses comparable with more traditional geometric morphometrics but with less need for expert training, less labour, less chance of operator error and a higher spatial resolution. We used this pipeline to analyse the previously unexamined craniofacial phenotype of Dp1Tyb mice, a relatively new model of DS (Lana-Elola et al., 2016), and identified localised differences more precisely than was previously possible. The high resolution enabled local deformation density mapping, which bypasses some of the issues around global scaling for shape comparison and enabled mapping of the dysmorphology specifically to the occipital and naso-palatal regions. We also applied the method to a second and larger dataset of outbred mice and showed it to be similarly effective for analysis of natural shape variation associated with size (allometry) and sex.

Both traditional and landmark-free methods revealed that Dp1Tyb mice have size and shape differences compared with WT mice that parallel the DS phenotype in previously described DS mouse models and in humans. Both methods separated Dp1Tyb mice from WT mice in shape space using one or two principal components and both revealed the significantly reduced size of the cranium and mandible of Dp1Tyb mice and, more specifically, both described brachycephaly (shortened head), resolved in the analysis to an overall size reduction plus cranial doming.

The dysmorphologies found in Dp1Tyb mice are similar to those found in Dp(16)1Yey mice, which contain an additional copy of the same Hsa21-orthologous region of Mmu16 (Starbuck et al., 2014). Specifically, the brachycephaly, midfacial hypoplasia and palatal phenotypes are present in both models. However, there are differences between the reported phenotypes. Some of these arise from the use of different landmarks. For example, the analysis of Dp(16)1Yey mice did not use any landmarks around the zygomatic arches and thus did not capture the relatively wider arch positions that we observed in Dp1Tyb mice. However, some of the phenotypic differences may be caused by other factors. The Dp(16)1Yey mice were bred on a mixed genetic background (C57BL/6J×C3H/HeJ F1) whereas we analysed Dp1Tyb mice on an inbred C57BL/6J background. Furthermore, although we analysed Dp1Tyb mice at 16 weeks of age, the age of the Dp(16)1Yey mice was not specified and thus could have been different. Finally, we have found that the crania and mandibles of genetically identical mice raised in different mouse facilities can be distinguished morphometrically using the landmark-free method we describe in this paper (Y.R., V.L.J.T., J.B.A.G., unpublished), indicating another potential source of variation between our study of Dp1Tyb mice and the previous analysis of Dp(16)1Yey mice.

The landmark-free method we have presented here is in some ways related to a much older method of shape comparison: D'Arcy Thompson's deformation grids, which were profoundly influential in the development of morphometrics (Bookstein, 1977; Thompson, 1942). Thompson's approach was landmark-free as it relied on deformation of a grid superimposed on an organism rather than points defined on the basis of anatomical features. Although such approaches have found traction in the neuroimaging community, they have not been adopted within geometric morphometrics aside from serving as inspiration or as heuristic foundation for key methods such as thin-plate splines. This is a major difference, as landmarks contain assumptions about what is important and require the underlying assumption of homology of landmarks across individuals or developmental stages (Bookstein, 2005), whereas deformation grids are an attempt to capture the entirety of morphological variation. The assumption of homology can be particularly problematic in studies of embryonic development in which anatomical features and tissues of origin may dissociate during morphogenesis (Hallgrímsson et al., 2015) and choices of landmark definition can make analyses blind to unexpected findings. Here, we have applied and validated a morphometric pipeline directly inspired by Thompson's grids and we have found that the method compares favourably with geometric morphometric analyses of craniofacial variation in mice.

One of the potential limiting factors of the landmark-free method is computational time. The computer used here for the majority of the analysis (iMac Pro 2017, 3 Ghz Intel Xeon W, 128 GB RAM) for 20 specimens (10 of each genotype) took ∼10 h to compare skulls with 2500 control points and ∼6 h for the mandible with 700 control points. For comparison, a Macbook Pro laptop (2.2 GHz Intel core i7, 16 GB RAM) took over ∼100 h to run a mandible analysis at 330 control points. For the DO analysis, it took ∼5 h to run 95 scans on a Linux (Ubuntu 20.04) workstation running an i7-9700 9th generation processor (8-Core/8-Thread, 12 MB Cache) with 64 GB RAM and a Dual NVIDIA GeForce RTX 2080 graphics cards with 8 GB VRAM. By contrast, full volumetric registration is much more computationally intensive, requiring access to high performance computing. A similar registration for the full volumetric scans for the DO sample using the MINC nonlinear registration pipeline would take weeks to run on a local workstation.

As well as being less labour-intensive, the landmark-free method has three additional advantages. The first is consistency: the landmark-free method overcomes the inter- and intra-operator error associated with manual placement of landmarks (Robinson and Terhune, 2017). The second is that less training and skill is needed: although the landmark-free method requires some manual input in the early stages, particularly in determining image thresholding and in cleaning up imperfect anatomical segmentation, it requires less user training than the landmark-based approach. Third, and most scientifically novel, is resolution: the landmark-free method provides much higher resolution and information density than the landmark-based method. In principle, the landmark-free method offers arbitrarily high resolution. In practice we found that decimating the initial mesh from ∼2,800,000 to ∼19,000 vertices for the cranium (∼200,000 to ∼16,000 for the mandible) and using a kernel size in the Deformetrica algorithm of 1 mm to yield ∼2500 control points for the cranium (∼700 for the mandible) captured the interesting anatomical features at high density while avoiding noise, e.g. trivial surface texture differences. Different sizes of specimen will have different optimal spatial parameters. The high density of control points was further refined by having them clustered algorithmically by Deformetrica at regions of high variability between samples. This might be contrasted with the inherent bias in landmarking that tends to place shape differences close to landmarks (observable in, for example, Fig. 3A,B). It can also be contrasted with the use of semi-landmarks, an approach which adds landmarks that are evenly distributed across contours or surfaces that are bounded by identifiable landmarks (Adams and Collyer, 2018; Gunz et al., 2005) but still potentially leaves gaps where landmarks are sparse. A trade-off for all methods that increase the number of discrete observations, however, is the increasing risk of overfitting as the number of variables (e.g. landmarks or voxels) increases relative to the statistical degrees of freedom, or the ‘curse of dimensionality’ (Indyk and Motwani, 1998). Controls for overfitting, such as the permutation test we applied, are therefore essential.

Is the higher resolution and more complete coverage useful? We found that the landmark-free approach allowed us to see changes not visible using the landmark-based approach. Most strikingly, we were able to observe a shape difference in the lower-posterior mandible, where landmarks are absent, and in the snout and palate, where landmarks are more abundant but possibly not dense enough to capture the localised in-plane differences. These latter changes in particular indicate homology with the mid-face hypoplasia found in humans with DS. This will be useful in understanding how DS genes result in dysmorphology because we now have a better knowledge of their location of action.

At another level, high resolution is useful because it enables mapping of surface ‘stretch’ by retaining all the vertices of the mesh, in effect making each vertex a landmark. The local nature of this deformation mapping makes it easier to interpret the visual display of the deformations without global scaling. The very short spatial scale of this mapping is likely to be a much better way to capture and localise changes. As these may be caused by alterations in different biological processes, such as cell proliferation or extracellular matrix expansion, analysis of which requires distinct analytical techniques, mapping changes to small areas using high-resolution morphometrics, makes efficient application of those techniques much more feasible. In contrast, the landmark-based method reveals net displacement across a broad area, where the underlying cause could be hundreds of cell diameters away.

Although the landmark-free method was developed for MRI scans of brains (Durrleman et al., 2014), it can in principle be applied to any 2D or 3D dataset for which a contour or surface can be defined. This could be anything from a manually-drawn contour to a well-segmented confocal microscopy image. Applying it to a mutant skull has enabled two important conclusions. The first is that using a landmark-free approach is still advantageous even when traditional landmarking is possible. The second conclusion is that it is possible to apply this approach in the form of a relatively user-friendly tool. We have found it useful in understanding the DS craniofacial phenotype but, with modest computational expertise, other researchers can tackle any mutant phenotype, including where traditional methods have struggled, such as in early developmental stages or other biological forms that lack well-defined landmarks.

## MATERIALS AND METHODS

### Mice and imaging

C57BL/6J.129P2-Dp(16Lipi-Zbtb21)1TybEmcf (Dp1Tyb) mice (Lana-Elola et al., 2016) were bred at the MRC Harwell Institute. All mice were backcrossed to C57BL/6JNimr for at least 10 generations. All animal work was approved by the Ethical Review Panel of the Francis Crick Institute and was carried out under project licences granted by the UK Home Office. Heads from 20 mice at 16 weeks of age were used (10 Dp1Tyb and 10 WT, five male and five female of each genotype). However, four subjects were excluded from the analysis owing to fractures in either the mandible or skull. Heads were prepared for µCT by fixation in paraformaldehyde and then scanned at a 25 µm resolution using a µCT 50 (Scanco). DO mice (Katz et al., 2020) were bred at the Jackson Laboratory. DO mice are specimens derived from eight inbred founder lines that included three mouse subspecies, resulting in a population with high genetic diversity. Heads from 98 DO mice at 12 weeks of age were used (72 female and 26 male). All of the DO scan data are available through the Facebase hub as project Record ID 1-731C (https://doi.org/10.25550/1-731C) (Hochheiser et al., 2011; Samuels et al., 2020).

### Landmark acquisition

Three-dimensional locations of 68 anatomical landmarks for the cranium (Fig. S1) as previously defined by Hallgrímsson et al. (2007) were placed onto 3D reconstructions of µCT images of DO, Dp1Tyb and WT mice, using either Microview (Parallax Innovations) or Analyze 3D software (AnalyzeDirect). We also placed 17 landmarks for the mandible of Dp1Tyb and WT mice (this was not carried out for the DO specimens as the latter analysis was for variance and validation purposes only). All landmarks that were placed manually were verified by checking orthogonal planar views of the subject. We also generated another set of 68 skull landmarks for the DO dataset, based on machine-learning enhanced whole-volume nonlinear registration as in Percival et al. (2019) and Devine et al. (2020), in which details of the method are given.

### Landmark-based shape difference analyses

#### Validation of landmark-free quantification of shape variation

For the Dp1Tyb and DO datasets, we quantified both shape distances and significance in shape differences between genotypes. Landmarks were aligned using a Generalised Procrustes Superimposition Analysis (GPA; Gower, 1975), and distances between landmarks for each subject were analysed by PCA using MorphoJ (Klingenberg, 2011) to visualise group separation by shape. To quantify significance in shape differences between genotypes we used the Procrustes Distance Multiple Permutations test (1000 iterations) within MorphoJ for the Dp1Tyb dataset and using the Geomorph package in R for the DO dataset. Centroid size was calculated as the square root of the sum of the squared distances from each landmark to the centroid, i.e. the centre of mass of all landmarks of a given specimen. The normalized centroid size was calculated by dividing the centroid size by the number of landmarks (or mesh vertices in the landmark-free method, see below) and was used to compare size differences between WT and Dp1Tyb crania and mandibles. The statistical significance of such size differences was calculated using a two-tailed unpaired *t*-test. Finally, we visualised shape variation with heatmaps using the R package Morpho (Schlager, 2017). Specifically, we used the Morpho function tps3d, which applies a thin-plate spline method (Bookstein, 1989) on mean landmark sets of each genotype group to interpolate an average mesh for each of the analysis groups. The Morpho R meshdist function was then used to create the heatmaps. The function first calculates the distances of the reference meshes vertices to that of the target meshes, for both the Dp1Tyb and DO datasets. Then, using a previously proposed algorithm (Baerentzen, 2005), the distances were given a negative value if inside the reference mesh or a positive value if outside. A vector containing blue and red colour values was assigned to the negative and positive values, respectively (Schlager, 2017).

### Landmark-free morphometric analysis

As an alternative to landmark population comparisons, statistical analysis of anatomical shapes can be achieved using so-called atlas-based approaches, which consist of estimating an anatomical model (i.e. template) as the mean of a set of input shapes (rather than point clouds) and quantifying its variation in a test population as deformations. This was previously achieved in a reproducible and robust landmark-free manner by Durrleman et al. (2014). This approach bypasses a number of problems associated with mesh point-to-point comparison by representing deformation between shapes as the diffeomorphic transformation of flow fields, i.e. currents over the mesh surface. Currents are parameterised by a set of control points in space and initial velocities, or momenta. By means of a gradient descent optimisation scheme, the method is able to produce a statistical atlas of the population of shapes. An atlas refers to a mean template shape, a set of final control point positions and momenta parameterising the displacements between the templates to each initial individual shape. In the following sections we describe the different steps to achieve such analysis. Landmark-free computations were conducted on an iMac Pro (2017, 3 Ghz Intel Xeon W, 128 GB RAM) (Dp1Tyb study) and a Linux (Ubuntu 20.04) workstation running an i7-9700 9th generation processor (8-Core/8-Thread, 12 MB Cache) with 64 GB RAM and a Dual NVIDIA GeForce RTX 2080 graphics cards with 8 GB VRAM (DO studies).

The steps of the landmark-free analysis pipeline are described below (see Fig. 1, Table 1 and Supplementary Materials and Methods for more information).

#### Step 1. Image segmentation and clean-up

Despite the fact that images acquired using µCT show good bone contrast, they often include the presence of artificial objects (noise and debris in the specimen), small holes and cartilage that need be excluded in order to obtain consistently comparable final surface meshes. To extract the surface meshes, a series of image processing steps were applied. After a thresholding operation to extract the skull, ‘morphological opening’ and ‘closing’ were performed on the binary mask to remove internal cartilage structures (Fig. S2). Removal of spurious objects was achieved by clustering, categorising all of the connected components in an image by size and retaining only the largest component. Skulls were segmented using bone density to isolate the mandible (the density of which is higher than that in the rest of the skull). However, this segmentation of the mandible can happen improperly and may include parts of the temporal bone, which must be cleaned and removed manually. Mandible binary masks were parcellated from the rest of the skull binary mask semi-automatically using Watershed segmentation (Mangan and Whitaker, 1999). For the 98 DO skulls, we segmented a binary mask comprising only the crania on the atlas (mean) as previously described (Percival et al., 2019). This allowed us to propagate a binary mask to obtain a clean segmentation (without other structures with similar density values, e.g. mandibles) across all 98 specimens.

#### Step 2. Mesh generation and refinement

Meshes were produced using marching cubes on the binary masks using VTK open source software to obtain meshes of the DO skulls, and batch converted the resulting binary OBJ files to Ascii PLY meshes. A script for all of these steps is included in the GitLab repository (https://gitlab.com/ntoussaint/landmark-free-morphometry). The meshes were then cleaned using different basic cleaning operations (removed faces from non-manifold edges, removed duplicated vertices and faces, and merged close vertices), followed by a surface Laplacian smoothing (Vollmer et al., 1999), and finally decimated to 1.25% of their initial number of faces for the Dp1Tyb dataset (yielding 20,000 vertices) and 5% for the DO dataset (yielding 90,000 vertices), for which the variation was more subtle. This allowed us to reduce overall computational time while improving mesh quality and maintaining overall topology and anatomical features. We performed all these operations using a bash script that automated this process across all the meshes with a Meshlab filter script (Cignoni et al., 2008).

#### Step 3. Mesh alignment

The atlas construction necessitates the production of aligned meshes from the input µCT images as a pre-processing step. This could have been achieved in a number of different ways; however, for these datasets we chose to use a small number of manually placed landmarks. In principle at least four landmarks are needed for alignment of 3D objects, but in practice we used either five or six landmarks (at least two pairs far from the midline) to achieve the alignment. After manual landmarks were placed, alignment was implemented using a Procrustes technique (Gower, 1975) involving a rigid body-plus-scaling transformation model (similarity alignment) or a rigid body transformation without scaling (rigid body alignment). As we had gathered these initial alignment landmarks in different software for the WT and Dp1Tyb specimens and the DO specimens [Microview/Analyze3D and MINC (https://github.com/BIC-MNI/minc-toolkit-v2), respectively], we converted the TAG landmark files we gathered in MINC into MPS landmark files for subsequent analyses, using our *tag2mps* R function. We have included several conversion scripts in our repository (https://gitlab.com/ntoussaint/landmark-free-morphometry/-/tree/master/data/preprocessing/) that can generate MPS files from other widely used landmark formats, such as TXT, CSV, TAG, and landmarkAscii.

#### Step 4. Landmark-free atlas construction and deformation capture

As previously described (Durrleman et al., 2014), the atlas construction major hyper-parameters consist of the size of the Gaussian kernel used to represent shapes in the varifold of currents, denoted σ_W_, and the number of control points, denoted N_cp_. Control points can be thought of as unbiased landmarks, initially they are spaced evenly on a regular grid but move slightly towards areas of greatest variation. Thus, as much as possible of the shape change is captured in an unbiased manner. σ_W_ can be seen as the precision at which the shape deformation is described. N_cp_ denotes the sampling density in space. Larger kernel sizes would have given lower resolution and coarser deformations. We used a 0.5 mm kernel size for the Dp1Tyb specimens, giving the number of control points N_cp_ as 2990 for the cranium and 860 for the mandible. The DO analysis (cranium only) was run at two control point densities, 0.5 mm and 1.2 mm, giving 2808 and 560 control points, respectively.

We automated the generation of XML files for both the model file and the dataset file with two bash scripts. These include details on the atlas construction parameters and information on the subset of specimens that would be used in the atlas construction, respectively. We configured the core model options (configurable in the XML model file) by optimizing all options to allow high sensitivity while still allowing relatively fast computational times. We used the Keops kernel (PyKeops; https://pypi.org/project/pykeops/) as it performs better with larger datasets. Finally, as per the Deformetrica guide recommendations (https://medium.com/miccai-educational-initiative/a-beginners-guide-to-shape-analysis-using-deformetrica-fa9e346357b7) we performed all of our computations using a CUDA driver in order to run our analyses using the GPU (option configurable under gpu-mode within the XML model file) (see GitLab repository for details). It is worth noting that the pipeline still works well without CUDA (and therefore using the CPU), but by using the GPU through CUDA we were able to significantly shorten our computational times.

Atlasing was run with the noise parameter set to 1/20th the kernel size and the deformation kernel width was set to the same value as the atlasing kernel width. These values were arrived at by trial and error, in the first place by visually checking the output shapes for distortion and then looking for convergence over ∼30-40 iterations and checking for the distribution of the shape-distance parameter (Deformetrica residuals) as being continuously and normally distributed.

#### Step 5. Statistical analysis and morph generation

The atlas outputs provide a dense amount of information that can be used for various statistical analyses. Centroid size was calculated as the square root of the sum of the squared distances from each mesh node to the centroid of all nodes of a given specimen and divided by the number of mesh vertices to generate the normalised centroid size. Non-linear Kernel PCA with dimension 5 was applied to the set of momenta produced from the atlas of the population, in order to find the principal modes of variation of the entire population. The resulting output provided a way to compare these results with the landmark-based PCA analysis. We projected the subjects onto the feature space for comparison purposes. Such projection provides dense information of shape differences between the two sub-populations. Local magnitude of the momenta interpolated at the template mean mandible (or cranium) mesh point locations produce morphs between the means of groups and allow for additional qualitative interpretation of shape differences. These can be saved as videos (Supplementary Materials and Methods, E – ‘Animation of deformations between subgroups’ and ‘Generating videos’). Stratified k-fold cross-validation analysis was performed on the PCA data to evaluate the statistical power of classification between the two groups. Significance of the classification score was tested using a multiple permutations test at 1000 iterations (Ojala and Garriga, 2010). To assess overfitting, subjects were randomly partitioned into two groups (‘scrambled groups’) and PCA analysis performed to generate inter-group vectors. The distribution of the vector magnitudes was tested for normality using the Shapiro-Wilk test.

### Direct comparison of landmark-based and landmark-free methods

For comparison to the GM (landmark-based) results, we performed multiple multivariate regressions of shape (Procrustes coordinates, control-point centered momenta) on centroid size as implemented in Geomorph (https://protect-eu.mimecast.com/s/t-https://cran.r-project.org/package=geomorph).

### Code availability

Python, R and bash scripts and documentation for the landmark-free morphometric analysis are freely available on GitLab (https://gitlab.com/ntoussaint/landmark-free-morphometry) and their use is also described in Supplementary Materials and Methods.

## Supplementary Material

Supplementary information

Reviewer comments
