# Supplementary material for: A landmark-free morphometrics pipeline for high-resolution phenotyping: application to a mouse model of Down syndrome
Source: Development. 2021 Mar 12;148(18):dev188631. doi: 10.1242/dev.188631 (PMC7969589; doi:10.1242/dev.188631)

Supplementary Movies

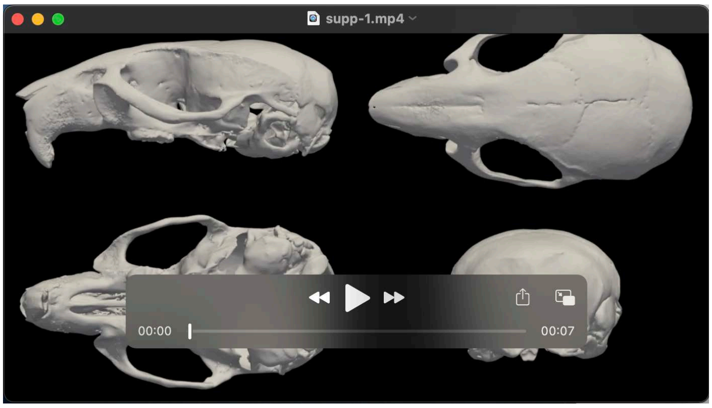

Movie 1.

Cranium morph including size

Cranium morph between the mean WT and Dp1Tyb shapes, where size has not been regressed out. Red and Blue colour change indicate expansion and contraction respectively as a percentage. Derived from landmark-free analysis.

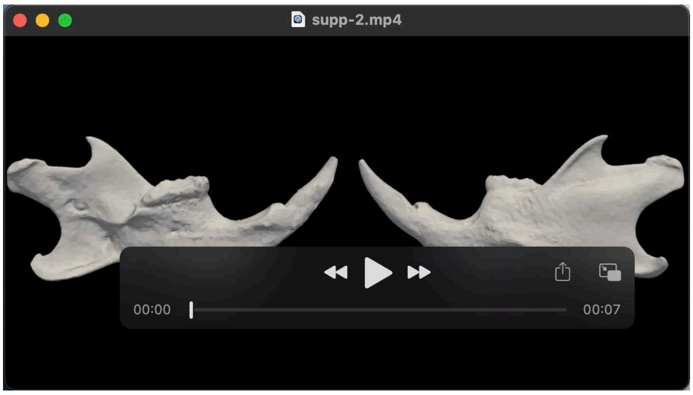

Movie 2.

Mandible morph including size

Mandible morph between the mean WT and Dp1Tyb shapes, where size has not been regressed out. Red and Blue colour change indicate expansion and contraction respectively as a percentage. Derived from landmark-free analysis.

Development • Supplementary information

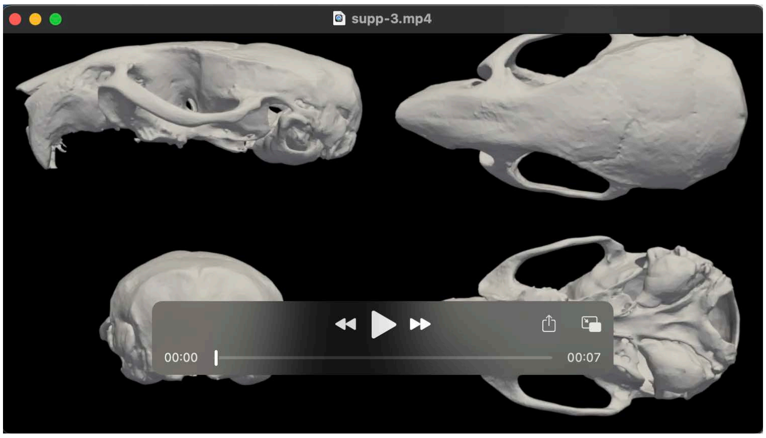

Movie 3.

Cranial morph displacement heatmap

Cranial morph between the mean WT and Dp1Tyb shapes, where size has been regressed out. Red colour change indicates the magnitude of displacement in mm. Derived from landmark-free analysis.

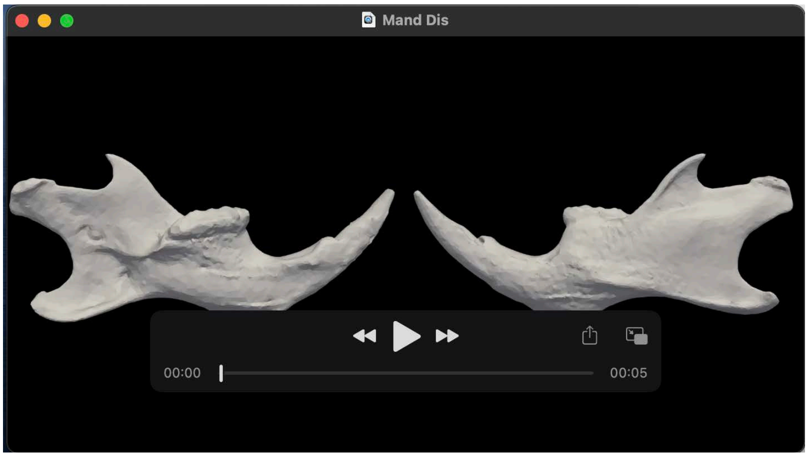

Movie 4.

Mandible morph displacement heatmap

Mandible morph between the mean WT and Dp1Tyb shapes, where size has been regressed out. Red colour change indicates the magnitude of displacement in mm. Derived from landmark-free analysis.

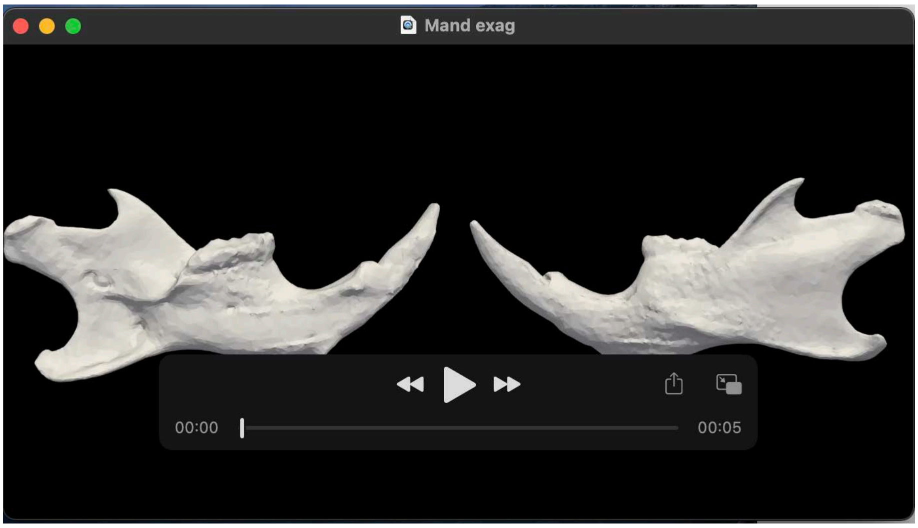

Movie 5.

Exaggerated Mandible Morph

Mandible morph between the mean WT and Dp1Tyb shapes, where size has been regressed out and the magnitude of the deformation has been exaggerated by a factor of 3 and so is not colour-coded. Derived from landmark-free analysis.

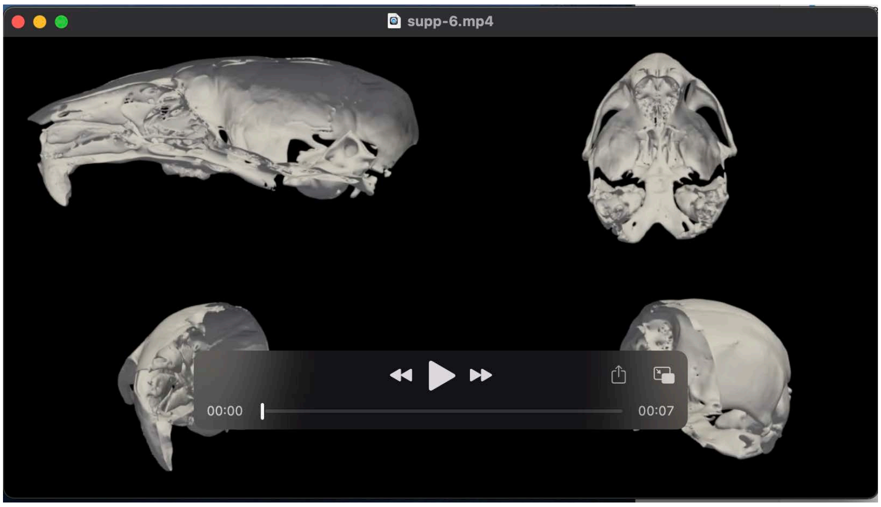

Movie 6.

Cranial Interior morph including size stretch heatmap

Cranial morph, showing the interior of the skull, between the mean WT and Dp1Tyb shapes, where size has not been regressed out. Red and Blue colour change indicate expansion and contraction respectively as a percentage. Derived from landmark-free analysis.

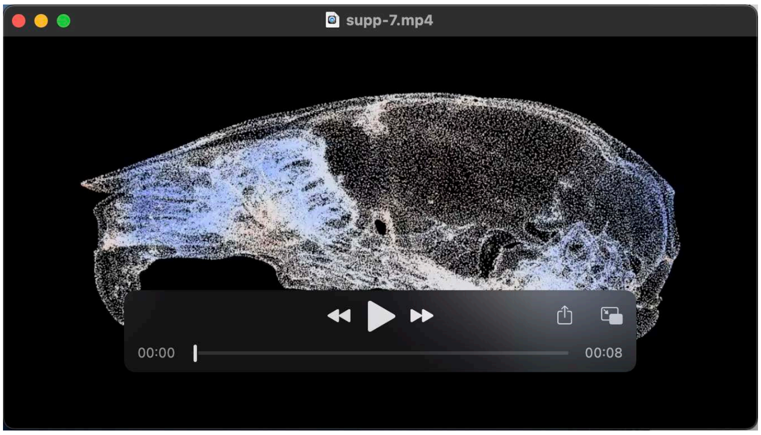

Movie 7.

Cranial Interior morph stretch heatmap

Cranial morph, showing the interior of the skull, between the mean WT and Dp1Tyb shapes, where size has been regressed out. Red and Blue colour change indicate expansion and contraction respectively as a percentage. Derived from landmark-free analysis.

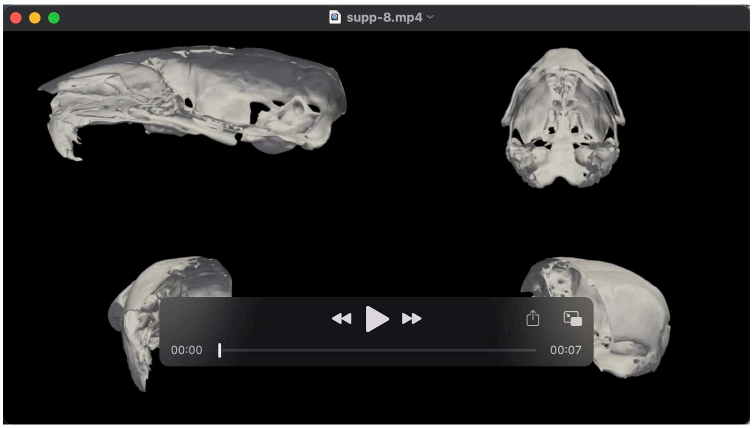

Movie 8.

Cranial mesh points stretch heatmap

Landmark-free-derived heatmap of cranium local stretch with no scaling. The mesh is represented here as all the points of the mesh rather than a rendered surface. Red and Blue colour change indicate expansion and contraction respectively. Downloading and viewing in a paused video by moving the slider is recommended.

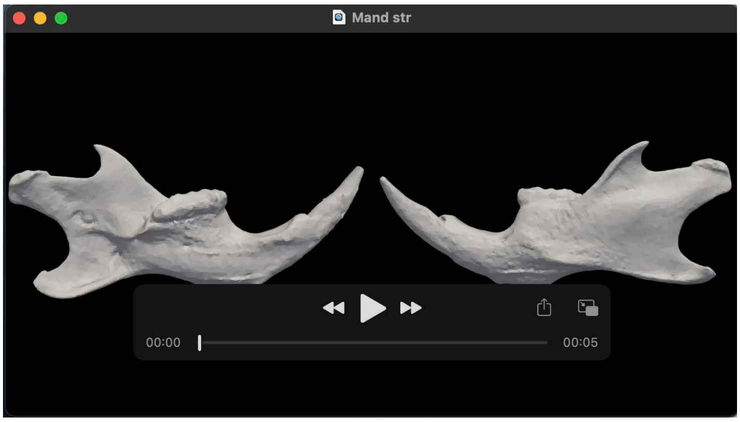

Movie 9.

Mandible morph stretch heatmap

Mandible morph between the mean WT and Dp1Tyb shapes, where size has been regressed out. Red and Blue colour change indicate expansion and contraction respectively as a percentage. Derived from landmark-free analysis.

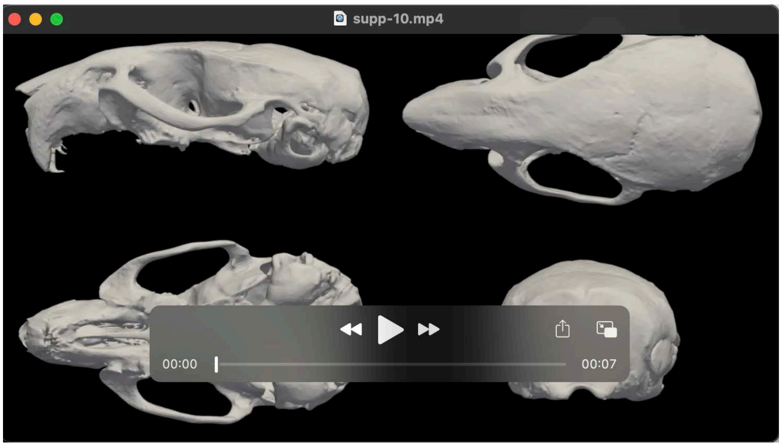

Movie 10.

Cranial morph stretch heatmap

Cranial morph between the mean WT and Dp1Tyb shapes, where size has been regressed out. Red and Blue colour change indicate expansion and contraction respectively as a percentage. Derived from landmark-free analysis.

Development • Supplementary information

Supplementary Figures

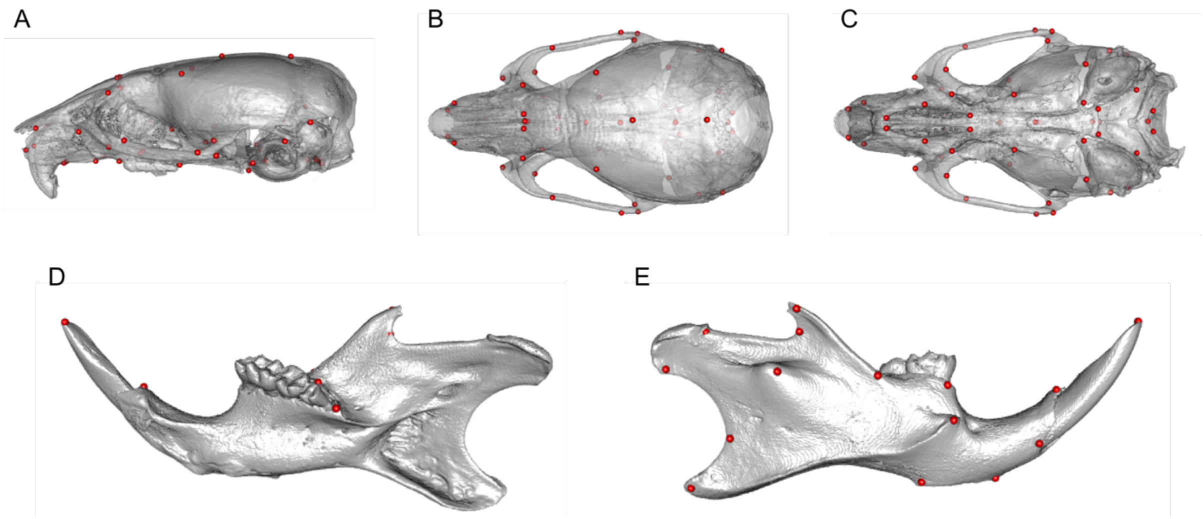

Figure S1.

Location of landmarks used in landmark-based morphometric analysis. A-E Anatomic locations of landmarks shown on a 3D digitized image of a µCT scan as previously defined [1,2]. 68 cranial landmarks are shown on lateral (A), superior (B), and inferior (C) views of the cranium. 17 mandibular landmarks are shown on lingual (D) or buccal (E) views of the mandible.

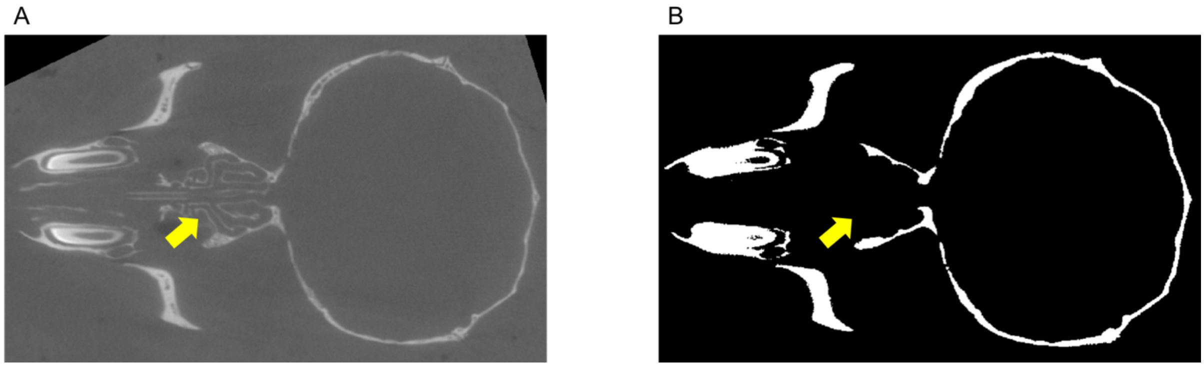

Figure S2.

Planar view of mouse head captured by µCT. A Image pre-processing. **B** Image post-processing. Yellow arrow indicates cartilaginous element (nasal turbinate).

Development • Supplementary information

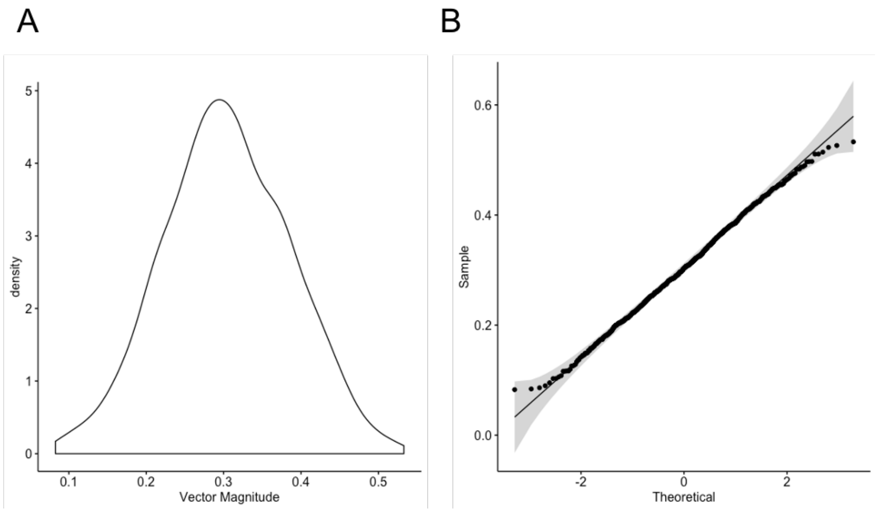

Figure S3.

Tests for overfitting show normally distributed data. A. Density plot and **B.** Q-Q plot show normally distributed data. Tests for normality conducted: Shapiro-Wilk ($p = 0.1587$) and Lilliefors (Kolmogorov-Smirnov) ($p = 0.258$).

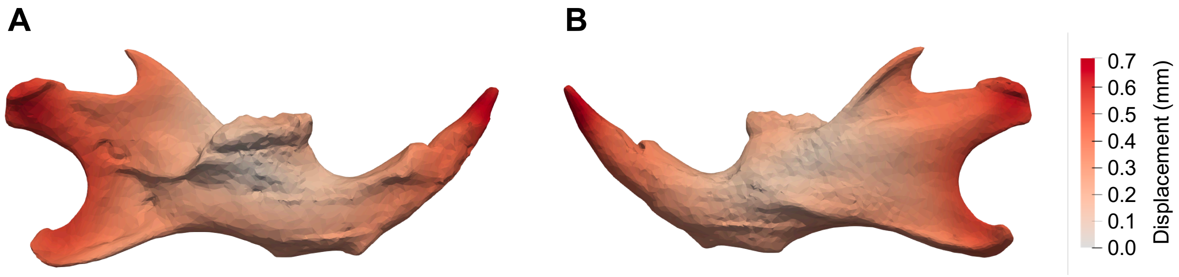

Figure S4.

Landmark-free displacement heat map with no scaling.

Without scaling, displacement changes are localised to the extremities.

Development • Supplementary information

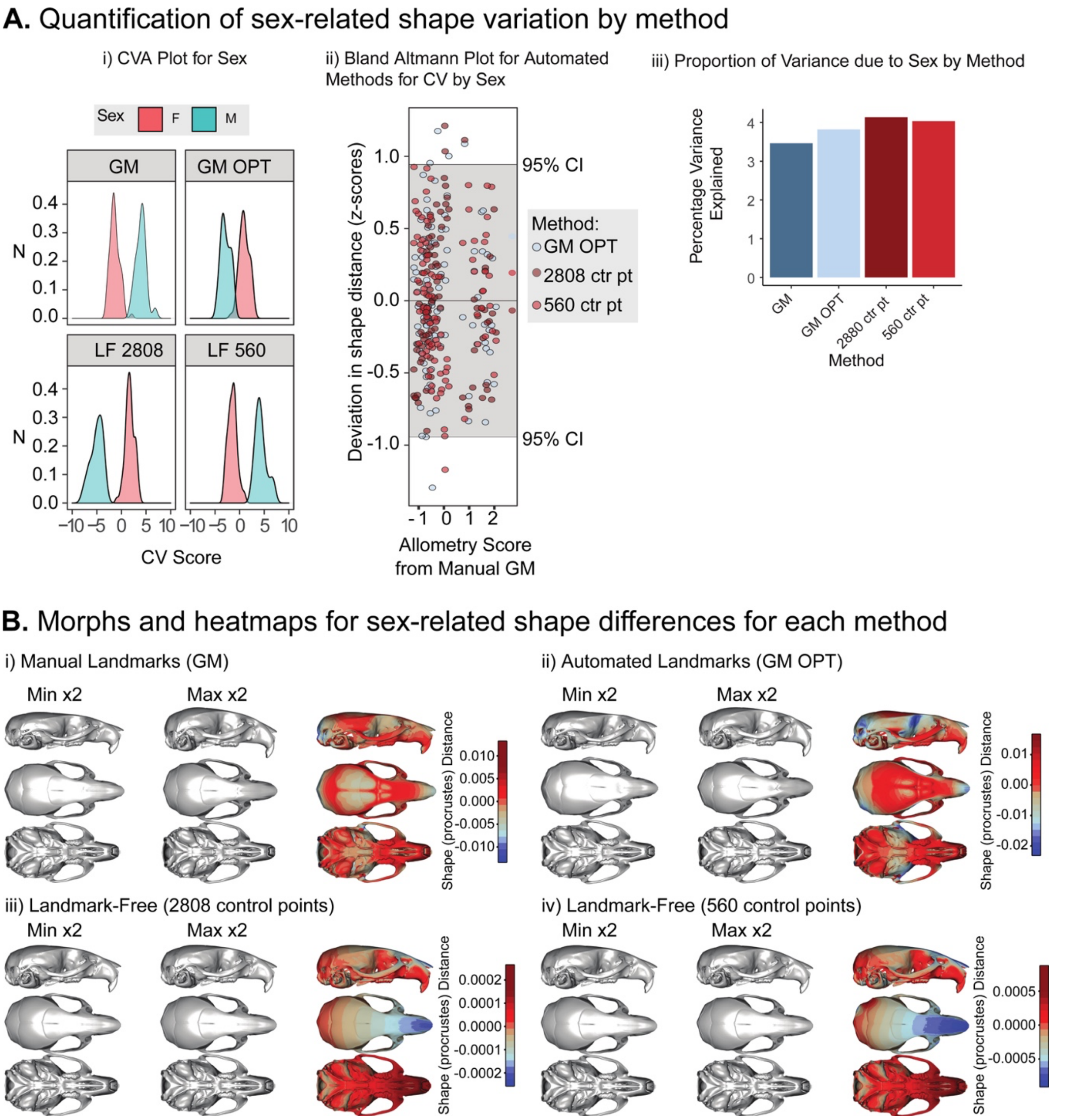

Figure S5. Validation of landmark-free morphometry in a sample of Diversity Outcross Mice by quantification of sexual dimorphism. **A.** Canonical Variance Analysis by sex; i) distributions of CV scores by method (unlike the DO group, these specimens are well-separated into two distinct groups); ii) Bland-Altman plot for CV scores for sexual dimorphism. Here, the deviations of each method from the manual landmarking-based estimated is plotted against the manual landmarking estimate; iii) variance components for sexual dimorphism and correlations among the CV scores across methods. **B.** 3D Morphs and heatmaps showing the shape variation associated with sexual dimorphism as estimated by each method. i) GM = manual landmark-based geometric morphometics; ii) GP OPT = automated landmark-based geometric morphometics; iii) LF 2808 and iv) LF 560 = landmark-free method with 2808 or 560 control points respectively. "Min x2" and "Max x2" label the deformations corresponding to the lower and higher ends of the heat map colour scales, exaggerated two-fold to more easily visualise the shape differences.

Development • Supplementary information

Supplementary Table

		Mean (±SEM) Normalised Centroid Size (mm)		Difference	
		WT	Dp1Tyb	Absolute	Percentage
Landmark-based	Cranium	6.387±0.061	5.939±0.061	0.448	7.01%
	Mandible	4.502±0.038	4.204±0.038	0.298	6.62%
Landmark-free	Cranium	6.987±0.063	6.514±0.063	0.473	6.77%
	Mandible	4.025±0.041	3.768±0.041	0.257	6.39%

Table S1.
Comparison of normalised centroid sizes of crania and mandibles from WT and Dp1Tyb mice determined using landmark-based or landmark-free methods.

Supplementary References

1. Hallgrímsson, B., Lieberman, D. E., Liu, W., Ford-Hutchinson, A. F. & Jirik, F. R. Epigenetic interactions and the structure of phenotypic variation in the cranium. *Evol. Dev.* **9**, 76–91 (2007).
2. Hallgrímsson, B., Dorval, C. J., Zelditch, M. L. & German, R. Z. Craniofacial variability and morphological integration in mice susceptible to cleft lip and palate. *J. Anat.* **205**, 501–517 (2004).

Development • Supplementary information

Supplementary Materials and Methods

Landmark-free Morphometrics Pipeline Description

This Appendix is intended as an overview of the landmark-free pipeline for a relative non-specialist. Detailed dcoumentation is embedded with the pipeline code published on GitLab at https://gitlab.com/ntoussaint/landmark-free-morphometry. This Appendix is organised into five sections as follows:

A. Pipeline Dependencies
B. µCT Pre-processing
C. Mesh Alignment
D. Atlas Construction
E. Shape Statistics

A. Pipeline dependencies and notes on initialisation

All packages and software listed below are required. The pipeline is written in Python and C++ and requires a suitable environment to run both. We used the browser-based Jupyter environment (https://jupyter.org/). Specific requirements for each segment of the pipeline are listed at the appropriate page within the pipeline code but the complete list is as follows:

Absolutely required:

FSL - https://fsl.fmrib.ox.ac.uk/fsl/fslwiki (Image analysis tools)
Deformetrica - http://www.deformetrica.org/ (Atlasing tools)
Git - https://git-scm.com/ (Code version-control system)
ITK - https://itk.org/ (Segmentation and registration tools)
VTK - https://vtk.org/ (Visualisation tools)
CMake - https://cmake.org/ (For compiling ITK and VTK)
Python packages:

- Matplotlib
- Numpy
- SimpleITK
- Vtk
- Pandas
- Seaborn

Software we used but for which there may be similar alternatives:

Meshlab - http://www.meshlab.net/ (Mesh decimation)
Itksnap - http://www.itksnap.org/pmwiki/pmwiki.php (Image manipulation tool)
Paraview - https://www.paraview.org/ (Image visualisation tools)
Mitk - http://mitk.org/wiki/The_Medical_Imaging_Interaction_Toolkit_(MITK) (Landmarking tool for coarse alignment)

The pipeline code is downloaded as landmark-free-morphometry-master.zip or ~.tar. After decompressing the package and moving to its directory within the Terminal app (Apple, Linux or equivalent in Windows). Jupyter is started using the command **ipython notebook** which opens the pipeline in the default system Web browser. The pipeline is presented as a series of virtual "pages" which contain "**cells**" (sections) of two types. One type defines a function, the other applies those functions to the data. Cells can be run individually or as an automatic sequence (see Jupyter documentation for details). An example of a function-defining cell is below:

Development • Supplementary information

Short description of what the particular cell does and the inputs, outputs and adjustable parameters.

Black text is code and teal text is a comment on what the code does.

Mesh extraction

mesh extraction from a binary image using Marching cubes and VTK

```
def extract_mesh(parcellation_file, output_mesh_file, smooth_NIter=20, smooth_relaxation=0.6):
    ''' Extract a mesh from a binary image using vtk

    The method uses Marching Cubes in order to extract a mesh outlining a binary segmentation image
    The mesh is then smoothed in order to avoid the pixelization effect

    @arg parcellation_file: input binary image file
    @arg output_mesh_file: output vtk mesh file
    @arg smooth_NIter: number of iterations for the smoothing filter
    @arg smooth_relaxation: Relaxation factor for the smoothing filter

    @ret: output_mesh_file
    '''

    import vtk

    ## read the input binary image
    reader = vtk.vtkMetaImageReader()
    reader.SetFileName(parcellation_file)
    reader.Update()
    ## perform marching cubes
    mc = vtk.vtkImageMarchingCubes()
    mc.SetInputConnection (reader.GetOutputPort())
    mc.ComputeNormalsOff()
    mc.ComputeGradientsOff()
    mc.ComputeScalarsOn()
    mc.SetValue(0, 0)
    ## smooth the output mesh
    smoother = vtk.vtkSmoothPolyDataFilter()
    smoother.SetInputConnection(mc.GetOutputPort())
    smoother.SetNumberOfIterations(smooth_NIter)
    smoother.SetRelaxationFactor(smooth_relaxation)
    smoother.FeatureEdgeSmoothingOff()
    smoother.BoundarySmoothingOn()
    ## write the output to file
    writer = vtk.vtkPolyDataWriter()
    writer.SetFileName(output_mesh_file)
    writer.SetInputConnection(smoother.GetOutputPort())
    writer.Write()

    return output_mesh_file
```

B. μCT Pre-processing

This section describes the code used to extract and produce a mesh from an initial μCT image. The following cells can be run with default values to define the required functions:

Binary Segmentation
- Defines functions used in the object extraction

Parcellation
- Defines functions required for parcellation of binary image

Mesh Extraction
- Defines function used in generating a mesh from a binary image

Mirror mesh file
- Defines function used to mirror the mesh file

Display help functions
- Defines functions used to display images within the notebook

Generic Imports
- Imports Numpy

Using **ITK-Snap** save μCT images in the **NIFTI** (.nii.gz) format.

The following five **cells** need to be edited to change parameters and filenames before running (N.B. annotations describe the sections that can or need to be changed, along with a brief description of inputs, outputs and function/s of the cell).

Development • Supplementary information

Load data

Loads in the image file to be processed.

Defines current working directory. Set the folder in which outputs are stored. This should not need to be changed as the 'data' and 'preprocessing' folders are already within the file along with the scripts.

```
working_directory = os.path.join(os.getcwd(), 'data', 'preprocessing')
input_image = os.path.join(os.getcwd(), 'data', 'uCT-1.nii.gz')
arr = display_image(input_image)
_ = plt.hist(arr.flatten(), np.linspace(0,15000,100), density=True)
```

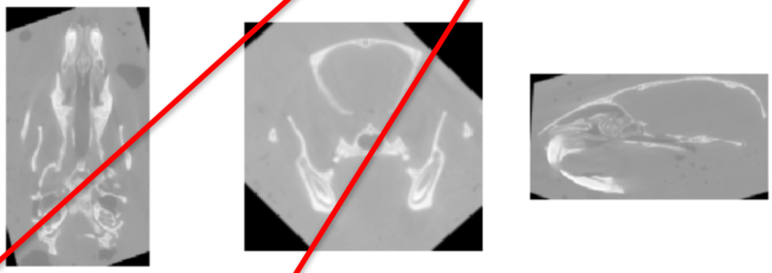

This tells the script where to look for the file to be processed. In this case within the **data** folder.

In this example uCT-1.nii.gz is the initial μCT image to be processed. This should be replaced with the filename desired.

Object extraction

The cell uses the functions defined in **object_extraction** to extract the object of interest from the input image and apply morphological functions to remove any noise and close unwanted holes.

Name of output file, a binarized image.

```
[9]: object_file = extract_major_object(input_image,
                                      os.path.join(working_directory,
     'uCT-1_object.nii.gz'),
                                      threshold=9000,
                                      closing_kernel=1,
                                      opening_kernel=0)

     arr = display_image(object_file)
```

Adjustable parameters
Threshold – an appropriate value should be selected to extract the region of interest from the original μCT image.
Closing_kernel and **opening_kernel** - parameters used to fill holes in the binary image. If none is required set to 0

Check output file using **ITK-Snap** to validate successful extraction of the region of interest. If the regions has been appropriately binarized it should be saved as **.mha** file.

Development • Supplementary information

Parcellation

This cell uses the functions defined in **parcellation** to ‘parcellate’ the image into various regions according to the density of the bone. This allows the separation of mandible (densest bone) from the rest of the skull. If no region separation is required skip this step and move to the **Mesh extraction** step:

Define the name for file to be segmented i.e. the file from **Object extraction**.

Watershed level should be .22 for full resolution image. (.1 is for the example data provided).

```
region_file = parcellate_object(object_file,
                                os.path.join(working_directory, 'uCT-1_regions.
  ↪mha'),
                                watershedlevel=.1,
                                shrink=1)

arr = display_image(region_file)
```

The output is a binary image with regions ordered by bone density. Use **ITK-Snap** to visualise these labels. The example on the right shows these regions, here label one is the red region. Note the label/s for region of interest (ROI).

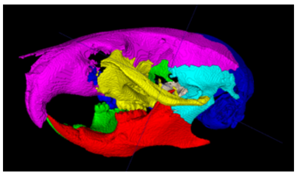

Object selection

Input the labels of relevance to separate the ROI from the rest of the subject.

Define the file name for the selected region of interest. This is the output file from **region parcellation**.

Input labels of regions to be extracted from the **region parcellation** step prior.

If only the first label is required, input 1 for both the lower threshold (l_threshold) and upper threshold (u_threshold). If the first 2 labels are desired set the values to 1 and 2 respectively. If the whole image is desired input the lowest and highest label numbers.

```
[11]: selectedobject_file = select_object(region_file,
                                          os.path.join(working_directory,␣
  ↪'uCT-1_selectedobject.mha'),
                                          l_threshold=1,
                                          u_threshold=1)

arr = display_image(selectedobject_file)
```

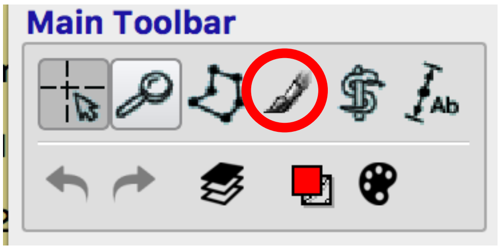

Check the output in **ITK-snap** opening it as a **segmentation file.** Any noise or irrelevant regions can be removed using the paint brush tool.

Development • Supplementary information

Extract mesh

This step uses functions defined in **Mesh Extraction** to extract a surface mesh from the region of interest selected prior. The meshes generated are in the **.vtk** format.

Output mesh file name.

```
[12]: mesh_file = extract_mesh(selectedobject_file,
                            os.path.join(working_directory, 'uCT-1_regions.vtk'),
                            smooth_relaxation=.2)
      png_file = display_mesh(mesh_file,
                            os.path.join(working_directory, 'uCT-1_regions.png'))
```

Mesh files produced are large in size and increase computational time of the **Atlasing** step to unrealistic durations. To overcome this, meshes were decimated using **meshlab**.
Save meshes in the **.stl** (stereolithography) format using paraview. Open the mesh in meshlab and apply **quadric edge collapse decimation** fi and enter the percentage reduction desired (authors used 0.15).

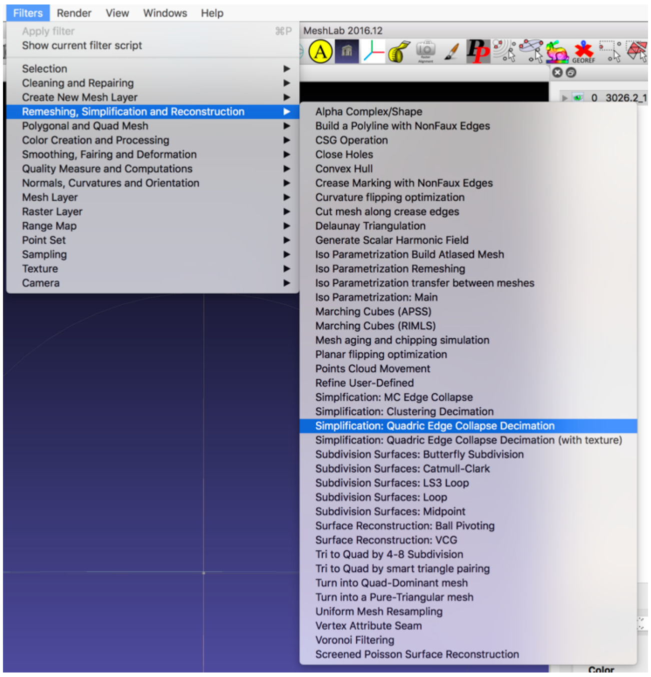

Once meshes for all subjects are generated move to the next step, **Mesh Alignment.**

Use paraview again to save the newly decimated mesh as a .vtk file and ensure it is saved in ASCII format

Development • Supplementary information

C. Mesh Alignment

This section describes the process of aligning the meshes produced in the previous section. Initial coarse alignment is required to allow the Atlasing process (below) to work. This first requires manually placing a small number of landmarks using **Mitk-Workbench**, minimally three (e.g. for a hemi-mandible) or more usually three symmetrically placed pairs. Landmark file names should be identical to the mesh file name e.g. Mandible_1.vtk, with its landmark file, Mandible_1.mps.
Below is shows landmarks used to align the mandible.

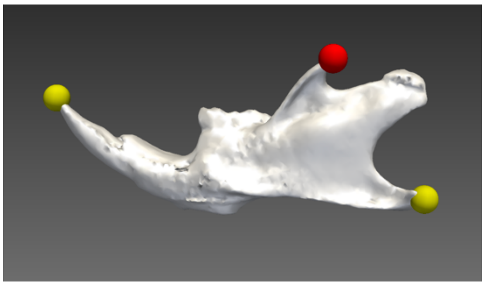

Once complete move landmark files (.mps) and mesh files (.vtk) into Alignment folder.
The following are run with default values:

Read landmarks

- Defines functions used to import landmark files

Numpy – vtk help functions

- Define functions used convert landmark files so they can be used to align meshes
- Functions used to extract centroid and centroid size

Procrustes alignment

- Defines functions used in alignment
    - Rigid – rigid body alignment
    - Similarity – rigid body plus scaling alignment

Load data

Loads meshes and associated landmark files ready for alignment,

Development • Supplementary information

Mesh Alignment

Uses functions defined in **Procrustes alignment** to align meshes. Two modes can be selected, **Similarity** or **Rigid body**. "Similarity" aligns meshes whilst regressing out the size. "Rigid body" aligns meshes but maintains size differences:

Choose mode here to '**Similarity**' or '**Rigid body'**

Adds a suffix to aligned mesh files so they can easily be distinguished from the original unaligned files.

```
[9]: output_meshes = align_meshes_to_center(meshfiles, landmarksfiles,
     mode='similarity', output_directory=working_directory, suffix='_r')

moving 1.vtk towards center with landmarks 1.mps -- determinant: 1.07
moving 2.vtk towards center with landmarks 2.mps -- determinant: 0.94
```

Mesh alignment fidelity should be checked in **Paraview**.

D. <u>Atlas Construction</u>

This section describes the application of the shape averaging and comparison (atlasing) processes.

Within the **atlasing** folder (included in the pipeline package) there are three files adjustable files:

Model.xml describes parameters for deformation:
– kernel-width: Order of magnitude of displacements for the template
– kernel-width: Order of magnitude for the subjects' displacements

```
model.xml
model.xml 〉 No Selection
<?xml version="1.0"?>
<model>
    <model-type>BayesianAtlas</model-type>
    <template>
        <object id="mandible">
            <deformable-object-type>NonOrientedSurfaceMesh</deformable-object-type>
            <data-sigma>10</data-sigma>
            <kernel-type>p3m</kernel-type>
            <kernel-width>2.0</kernel-width>
            <filename>initial_template.vtk</filename>
        </object>
    </template>
    <deformation-parameters>
        <kernel-width>1.5</kernel-width>
        <kernel-type>p3m</kernel-type>
        <number-of-timepoints>10</number-of-timepoints>
    </deformation-parameters>
</model>
```

Here, define the folder in which the pipeline will search for inputs and store outputs. By default, it is set to the alignment folder within data.

```
[8]: %matplotlib inline

### General Imports
from glob import glob
import os

working_directory = os.path.join(os.getcwd(), 'data', 'alignment')

### Find meshes and landmark files
meshfiles      = glob('{}{}*.vtk'.format(working_directory, os.sep))
landmarksfiles = glob('{}{}*.mps'.format(working_directory, os.sep))

meshfiles[:] = [el for el in meshfiles if not (('initial_template.vtk' in el)
  or ('_r.vtk' in el)) ]

### assert similar size and sort lists
assert(len(meshfiles) == len(landmarksfiles))
meshfiles.sort()
landmarksfiles.sort()

%matplotlib inline
import os
from matplotlib import pyplot as plt
pngfilename = os.path.join(os.getcwd(), 'Images', 'unaligned-meshes.png')
plt.figure(figsize=(10,10))
plt.imshow(plt.imread(pngfilename))
plt.axis('off')
plt.show()
```


Development • Supplementary information

Optimization_parameters.xml describes parameters that drive the minimization procedure:
– max-iterations: Stop criterion for iterations, usually ~150
– number-of-threads: Number of threads to use, usually equal to the number of subjects (should not exceed the number of threads available on the system)

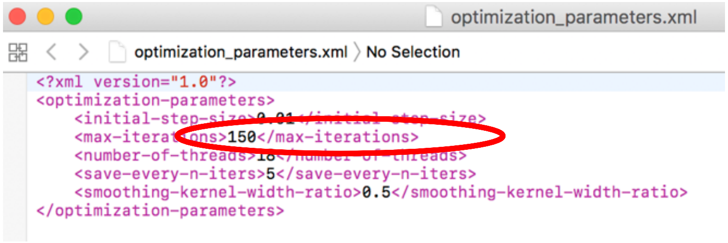

```
optimization_parameters.xml
optimization_parameters.xml 〉 No Selection
<?xml version="1.0"?>
<optimization-parameters>
    <initial-step-size>0.01</initial-step-size>
    <max-iterations>150</max-iterations>
    <number-of-threads>18</number-of-threads>
    <save-every-n-iters>5</save-every-n-iters>
    <smoothing-kernel-width-ratio>0.5</smoothing-kernel-width-ratio>
</optimization-parameters>
```

Data_set.xml defines the files to be atlased and should be structured as follows:

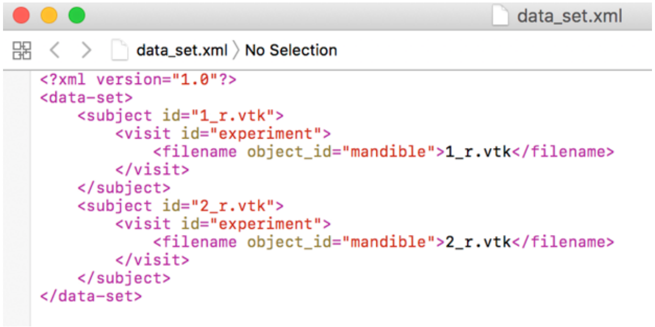

```
data_set.xml
data_set.xml 〉 No Selection
<?xml version="1.0"?>
<data-set>
    <subject id="1_r.vtk">
        <visit id="experiment">
            <filename object_id="mandible">1_r.vtk</filename>
        </visit>
    </subject>
    <subject id="2_r.vtk">
        <visit id="experiment">
            <filename object_id="mandible">2_r.vtk</filename>
        </visit>
    </subject>
</data-set>
```

From the alignment folder transfer aligned meshes (files with suffix **_r**) and initialtemplate.vtk into the atlas folder.

Deformetrica

The first cell loads the **Deformetrica** software. If the software is not found an error is returned.

```
[ ]: def which(program):
        ''' Search for a program on disk
        '''
        import os
        def is_exe(fpath):
            return os.path.isfile(fpath) and os.access(fpath, os.X_OK)

        fpath, fname = os.path.split(program)
        if fpath:
            if is_exe(program):
                return program
        else:
            for path in os.environ["PATH"].split(os.pathsep):
                exe_file = os.path.join(path, program)
                if is_exe(exe_file):
                    return exe_file

        return None

    deformetrica_location = which('deformetrica')
    if deformetrica_location is None:
        print('deformetrica error: Please install the deformetrica library: www.
     ↪deformetrica.org')
    else:
        print('Found deformetrica at this location: {}'.
     ↪format(deformetrica_location))
```

Launch Simulation

Launches the atlasing software using the files and parameters defined by model.xml, optimisation_parameters.xml and data_set.xml.
Deformetrica.log, within the output folder, contains information on the progress of the atlas, including the number of control points and the current iteration.

Development • Supplementary information

```
[ ]: import os

working_directory = os.path.join(os.getcwd(), 'data', 'atlasing')
script_filename = os.path.join(working_directory, 'deformetrica.sh')
os.system('bash {}'.format(script_filename))
```

E. Shape statistics

This section describes the statistical analysis of the outputs from the atlas.
Atlasing generates several output files of which the following three are used for statistical analysis:

- **Atlas_controlpoints.txt**
    - Control points of the atlas
- **Atlas_momenta.txt**
    - Momentum vectors of the atlas
- **Atlas_initial_template.txt**
    - Average mesh of the population

These files are must be moved into the **shape statistics** folder. In the same folder, a user-populated file **data.csv** defines the names and types of the subjects.

data.csv example file
In this example in GroupId, 1 refers to a mutant and -1 to WT, and in Gender, 1 Male and -1 female.

	A	B	C
1	id	GroupId	Gender
2	33.4a	-1	-1
3	33.4b	-1	-1
4	35.1c	-1	-1
5	37.1c	1	1
6	38.1c	-1	1
7	29.3d	-1	1
8	29.3g	-1	1
9	30.3b	-1	-1
10	30.3d	1	-1

The following cells are the run with default values:

Imports

- Loads relevant packages required for this section of the pipeline

Deformetrica

- Loads deformetrica ready to be used (required for creation of morphs)

Load data

- Loads output files stated previously to be analysed

Define population's groups

- Loads data.csv

Development • Supplementary information

Subgroup definitions

- Assigns names to groups of specimens defined by one or more parameters in **data.csv**.

[Default groups are: WTf (Wildtype female, defined by: group ID = -1, gender = -1, CRf is defined by: group id = 1, gender =-1), WTm (Wildtype Male), CRf (Carrier female) and CRm (Carrier Male)]
However, the user should change the names and definitions of subgroups to suit their needs.

```
In [48]: g1 = df['GroupId'].values < 0
         g2 = df['GroupId'].values > 0

         WTf = list(df.loc[(df['GroupId'] < 0) &
                           (df['Gender']  < 0)].index)
         WTm = list(df.loc[(df['GroupId'] < 0) &
                           (df['Gender']  > 0)].index)
         CRf = list(df.loc[(df['GroupId'] > 0) &
                           (df['Gender']  < 0)].index)
         CRm = list(df.loc[(df['GroupId'] > 0) &
                           (df['Gender']  > 0)].index)
```

Kernel Principal Component Anaylsis (kPCA)

This cell runs a principal component analysis (PCA) and produces a classification score (as a percent) and a p value for said score. As well as defining group IDs (by default group one > 0 and group two < 0 using values set in **data.csv**) and calculating the mean Principal Component score.

Development • Supplementary information

Cross validation score and standard deviation

Cross validation score and p value of permutation test

```
SVM Classification score 0.90 +/- 0.07)
/Users/yushi/anaconda3/lib/python3.6/site-packages/sklearn/model_selection/_split.py:598: FutureWarning: You should s
pecify a value for 'n_splits' instead of relying on the default value. The default value will change from 3 to 5 in v
ersion 0.22.
  warnings.warn(NSPLIT_WARNING, FutureWarning)
Classification score 0.90 (pvalue : 0.0010)
<Figure size 504x360 with 0 Axes>
```

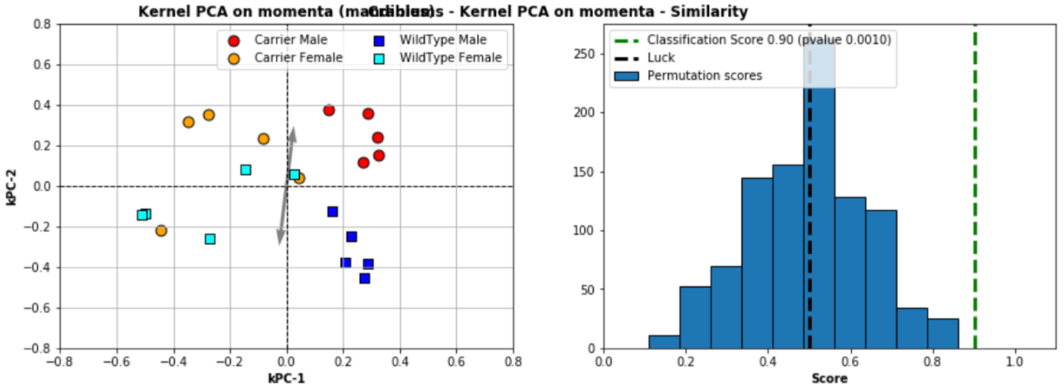

Eigenvalues/Eigenvectors

Produces Eigenvalues and eigen vectors. Providing data on how much variability each principle component describes.

Write PCA points on disk

Produces an excel file, kpca.csv containing the PC values which can exported to produce PCA graphs.

Momenta projection

Produces momenta that describes project between the mean of the whole population to the means of the previously defined populations.

Shoot mean shape between groups

Uses the momenta projection to ‘shoot’ the shape towards the means of the two groups.

Shooting outputs

Saves the shot meshes produced in the previous step in two locations:

- data/shapestatistics/shooting/forward
- data/shapestatistics/shooting/backward

Development • Supplementary information

They correspond to the mean shape (**Atlas_initial_template.vtk**) displaced towards the mean of group one (backward) and the mean of group two (forward). Each file now contains several meshes. The first mesh in the folder is the average shape of the whole population and the last mesh the mean shape of the subgroup. Each mesh in-between represents a step of deformation moving from one to the other.

Animation of deformations between subgroups

Reorders the mesh files produced in **Shooting outputs** to generate an animation of cyclic shape change starting with the mean of group one to the mean of group two and back again. The files for the cyclic deformation animation are saved in: data/shapestatistics/shooting/combined. Files should be opened in **Paraview** to visualise the meshes.

The deformation is viewed as a cycling animation by pressing the repeat and play buttons.

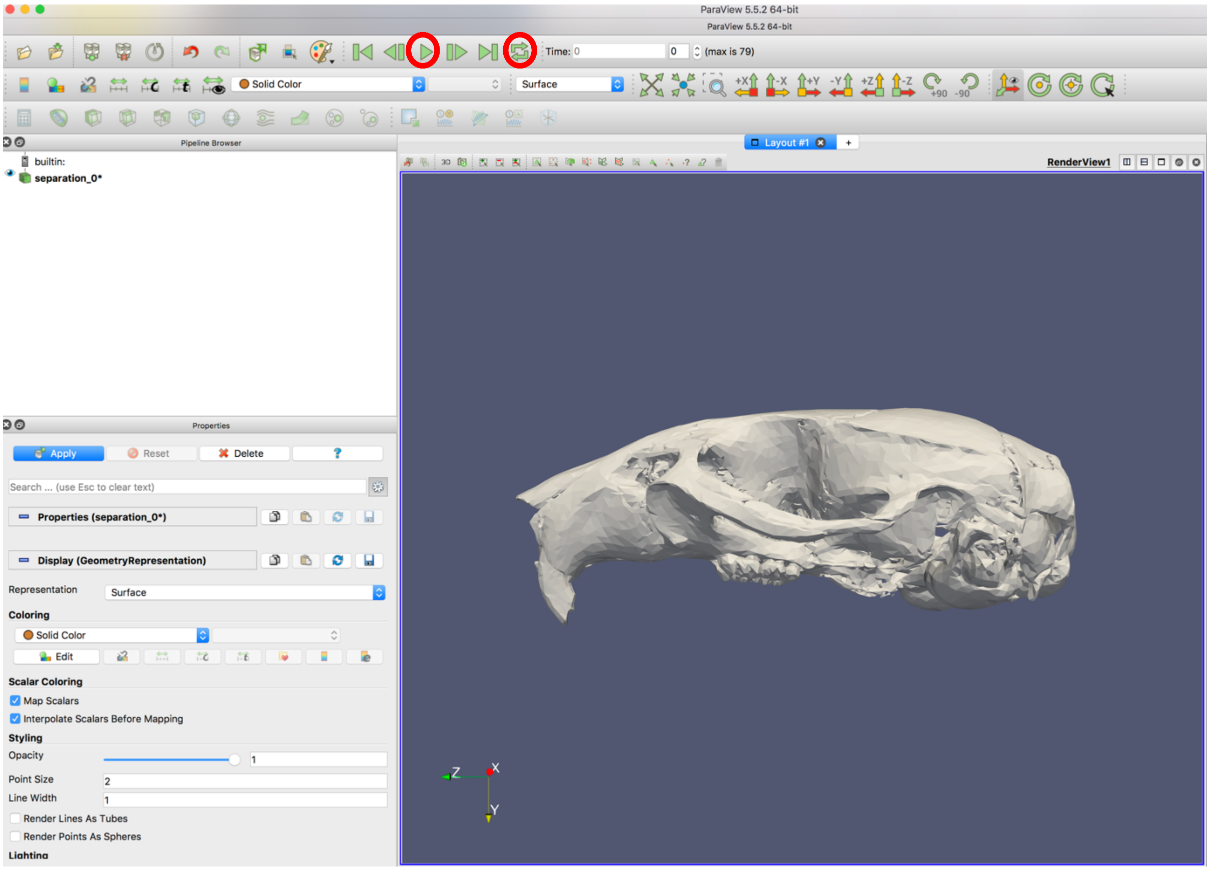

"Stretch" map (Local volume change map) between groups

This step integrates data on displacement and stretch into the cyclic deformation produced in the previous step. Removing the # from in front of **compute_point_displacements** calculates displacement.

```
    compute_point_displacements(m, mean, m)
    #compute_cell_surfaces_differences(m, mean, m)
    #compute_absolute_cell_surfaces_differences(m, mean, m)
    ### write mesh
    writer = vtkPolyDataWriter()
    writer.SetInputData(m)
    writer.SetFileName(mfile)
    writer.Update()

cyclic_combination(meshfiles1, meshfiles2, os.path.join(shooting_directory, 'combined'))
```

Removing the # from in front of **compute_cell_surfaces_difference**, calculates stretch.

```
    #compute_point_displacements(m, mean, m)
    compute_cell_surfaces_differences(m, mean, m)
    #compute_absolute_cell_surfaces_differences(m, mean, m)
    ### write mesh
    writer = vtkPolyDataWriter()
    writer.SetInputData(m)
    writer.SetFileName(mfile)
    writer.Update()

cyclic_combination(meshfiles1, meshfiles2, os.path.join(shooting_directory, 'combined'))
```

Heat maps are viewed by importing the mesh files from the combined folder into **Paraview**. Select displacements or Absolute Volume from the colouring drop down menu and the morph was played as before to visualise the heat maps. Colour scale (look-up table) may need to be adjusted to visualise differences properly. This can be found by clicking the edit button found underneath the dropdown menu.

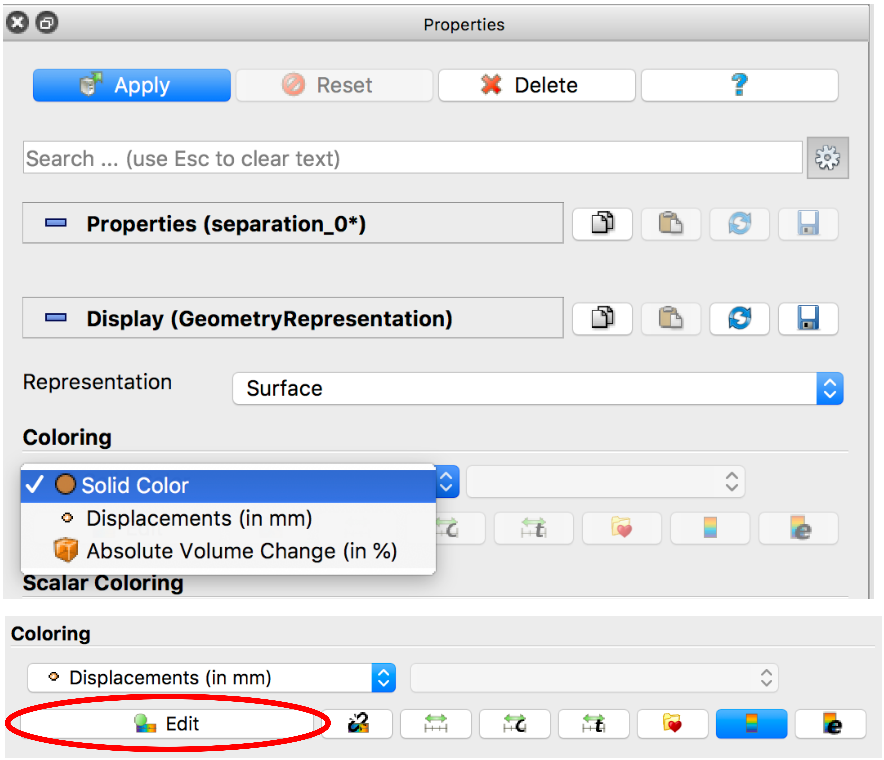

Development • Supplementary information

Generating videos

To save these morphs as videos (either with heat map colours or not). Go to file and save animation.

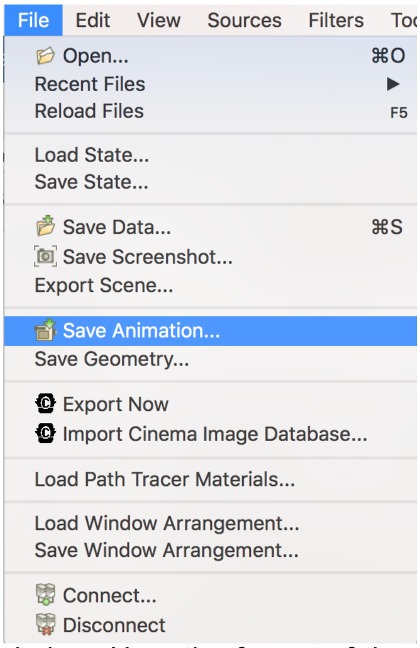

This will open up a file save window. Here the format of the video (.avi as default) and the name of the video can be chosen. This will bring up a second window in which a number of parameters for the video such as resolution and frames per second maybe adjusted.

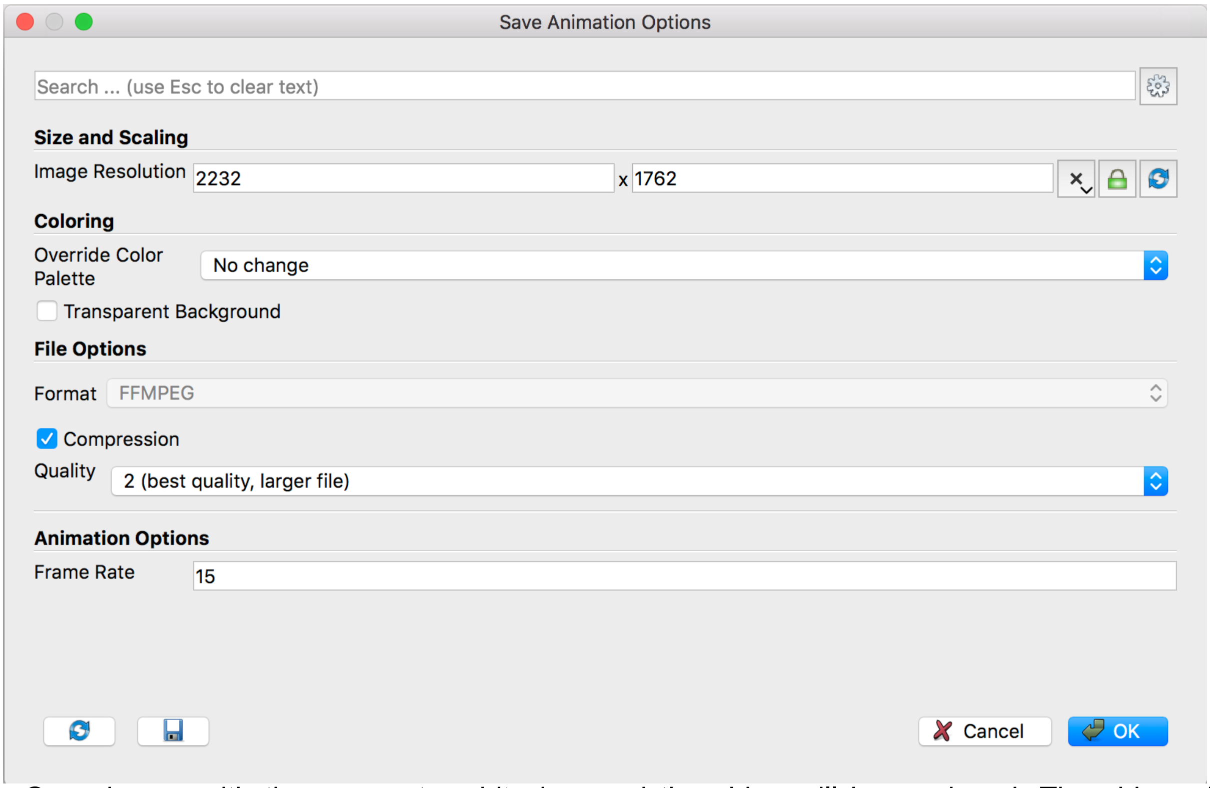

Once happy with the parameters hit okay and the video will be produced. The video will capture everything in the rendering window including colours scale bars. To edit the appearance of the scale bar, click the edit colour bar icon.

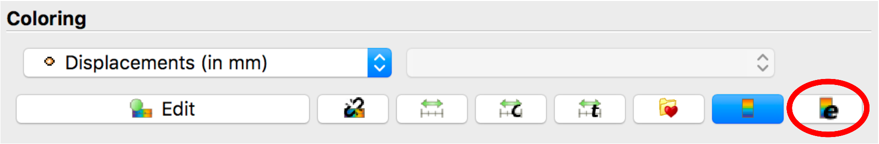

This will bring up a menu in which various settings of the colour scale bar may be changed.

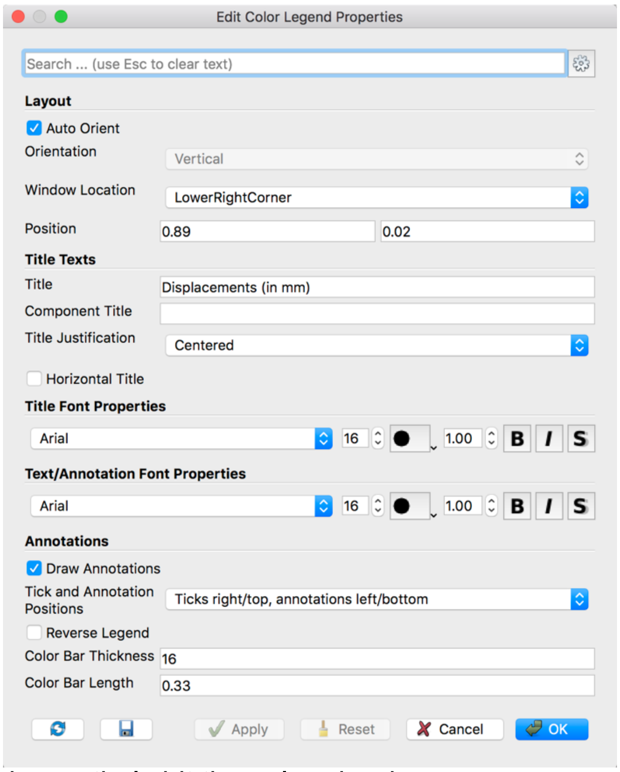

To remove the scale bar entirely hit the colour bar icon.

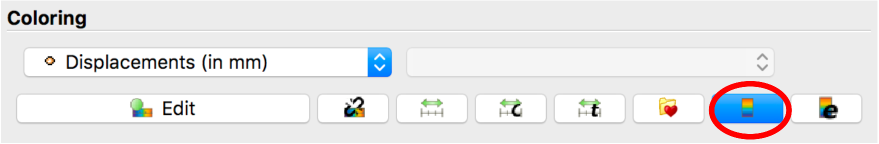

Supplement: Supplementary information [file develop-148-188631-s1.pdf]
