# Supplementary material for: A landmark-free morphometrics pipeline for high-resolution phenotyping: application to a mouse model of Down syndrome
Source: Development. 2021 Mar 12;148(18):dev188631. doi: 10.1242/dev.188631 (PMC7969589; doi:10.1242/dev.188631)
Supplement: Reviewer comments [file develop-148-188631_review_history.pdf]

A landmark-free morphometrics pipeline for high-resolution phenotyping: application to a mouse model of Down Syndrome

Nicolas Toussaint, Yushi Redhead, Marta Vidal-Garcia, Lucas Lo Vercio, Wei Liu, Elizabeth M. C. Fisher, Benedikt Hallgrimsson, Victor L. J. Tybulewicz, Julia A. Schnabel and Jeremy B. A. Green
DOI: 10.1242/dev.188631

Editor: John B Wallingford
Review timeline
Original submission: 23 January 2020
Editorial decision: 6 March 2020
First revision received: 2 December 2020
Accepted: 1 February 2021

Original submission

First decision letter

MS ID#: DEVELOP/2020/188631

MS TITLE: Application of high-resolution landmark-free morphometrics to a mouse model of Down Syndrome reveals a tightly localised cranial phenotype

AUTHORS: Nicolas Toussaint, Yushi Redhead, Wei Liu, Elizabeth Fisher, Benedikt Hallgrimsson, Victor Tybulewicz, Julia Schnabel, and Jeremy Green

I have now received all the referees' reports on the above manuscript, and have reached a decision. The referees' comments are appended below, or you can access them online: please go to BenchPress and click on the 'Manuscripts with Decisions' queue in the Author Area.

As you will see, the referees express considerable interest in your work, but have some significant criticisms and recommend a substantial revision of your manuscript before we can consider publication. In particular Reviewer #1 was the most critical and i share many of these concerns.

If you are able to revise the manuscript along the lines suggested, which may involve further experiments, I will be happy receive a revised version of the manuscript. Your revised paper will be re-reviewed by one or more of the original referees, and acceptance of your manuscript will depend on your addressing satisfactorily the reviewers' major concerns. Please also note that Development will normally permit only one round of major revision.

Please attend to all of the reviewers' comments and ensure that you clearly highlight all changes made in the revised manuscript. Please avoid using 'Tracked changes' in Word files as these are lost in PDF conversion. I should be grateful if you would also provide a point-by-point response detailing how you have dealt with the points raised by the reviewers in the 'Response to Reviewers' box. If you do not agree with any of their criticisms or suggestions please explain clearly why this is so.

© 2021. Published by The Company of Biologists under the terms of the Creative Commons Attribution License (https://creativecommons.org/licenses/by/4.0/).

Reviewer 1

Advance summary and potential significance to field

The method presented potentially provides a significant advance to the field. However, the presentation of the data is sloppy, making evaluation of the method difficult. Additional tests especially of population variance would improve the evaluation and application of the method.

Comments for the author

The article by Toussaint and colleagues describes new methods for landmark-free morphometric analyses of craniofacial shapes. A landmark-free method for morphometric analysis would be a great addition to the developmental biology community. In this manuscript, some promising data is presented, but further analyses should be included to provide a more thorough comparison of the technique to landmark-based methods, especially one that quantifies variance. Additionally, the figures lack critical information and are presented in inconsistent manners making comparisons and interpretations of the data challenging, if not impossible. This sloppy presentation detracts from a potentially interesting manuscript.

Major points:
1. The first section of the results “Landmark-based and landmark-free . . . “ is not a result. It is a summary of the methods, which they also describe in more detail in the methods. Its inclusion here seems unnecessary and confusing since critical information is lost in the summary. For clarity, I recommend removing this section.
2. In Figure 2, the graphs should be presented on similar scales to facilitate comparison. The PCA plots include different shapes and colors that are not described in the legend, making it impossible to interpret the data.
3. For the PCA analyses presented in Figure 2, there appear to be differences in population variance in the mandible samples (assuming I properly interpreted the shapes), with the landmark-free sample potentially exhibiting more variance in both WT and mutant mandible populations. As variance is an important measure of any population, any difference in the methods in reporting variance should be noted. Therefore, population variance should be quantified and any difference between the methods should be discussed. An accurate quantification of variance may involve increasing sample size.
4. In Figures 3 and 4 the scale for distance differs by an order of magnitude. In figure 4, the mandible distances go from 0.09 to 1.0 There is also only one scale for landmark-based methods and two for the landmark-free. The color code is also not the same on this distance scales, again making comparisons of the methods difficult.
5. The interpretation of the data presented in Figure 5 is that the mutant phenotype is “clearly . . . a growth deficit in the occipital region.” Given that the authors have only investigated one developmental stage and have not assessed proliferation, differentiation, or cell death of any tissues at any time, conclusions about growth cannot be made. Their stretch data may imply some size difference, but without reference to any developmental data, it is a real stretch to say it represents a growth difference. Further, there appear to be quite a number of areas in the skull that show differences in “stretch.” Why is the occipital region singled out? Do the authors have modeling data to show that differential growth in this region of the skull could mimic the shape changes in the DS mice? Overall, this data appears to be over-interpreted and any advantages of this technique are not clearly presented.

Minor points:
1. In the Introduction, the authors provide some details on previous work using the DS model mouse phenotypes. They say that Ts65Dn and Dp(16)1 Yey mice were shown to resemble the DS phenotype, and then go on to say that Dp1Yey mice demonstrate statistically significant dysmorphology. What is meant by this? Shouldn’t both mouse lines exhibit dysmorphology if they resemble the DS phenotype? Is this the more severe of the two lines? the less severe of the two? I gather the authors are trying to justify their choice to focus on this particular line but the reasoning is not clear. It seems that the best test of the method would be to use the less severe line. In any case, some rewording of this paragraph would help clarify why the specific model was chosen.

© 2021. Published by The Company of Biologists under the terms of the Creative Commons Attribution License (https://creativecommons.org/licenses/by/4.0/).

2. The authors should use a consistent terminology for the mice. In the introduction they use both Dp(16)1 Yey and Dp1Yey, while in the discussion they say Dp1Tyb, which I can only assume refers to the same mice.
3. Assuming Dp1Tyb refers to the same mice that they present data for in the paper, they say in the discussion that this mouse model was previously unexamined. Yet in the introduction (as noted in point 1 above), they describe previous work on this mouse.
4. Some references are listed as numbers 5.
The inclusion of "tightly localised cranial phenotype" in the title is misleading and also vague. the point of the paper is a method comparison, so it would be more appropriate to have some conclusion about the difference in the methods in the title.

Reviewer 2

Advance summary and potential significance to field

The manuscript by Toussaint et al., “Application of high-resolution landmark-free morphometrics to a mouse model of Down Syndrome reveals a tightly localised cranial phenotype” presents a landmark free methodology to quantify structures, with specific applications to craniofacial abnormalities. Landmarks are often difficult to place because a point may be hard to define or absent because of a disorder. Repeatability between investigators as they place landmarks can also be a problem. This methodology purports to overcome those and other barriers to quantify craniofacial structure. The authors compare landmark based and landmark free craniofacial analyses in the Dp1Tyb mouse model of Down syndrome. This landmark free methodology is touted to be accessible to biologists and geneticists who may not be as familiar with the more rigorous landmarking analyses.

Comments for the author

The following changes and additions would make this manuscript more suitable for publication:
Figure 2 is nearly impossible to read. Additionally, to accurately see the differences between the landmark free and landmark based approaches, the data should be presented on graphs with the same vertical axes. As presently constituted, Figure 2 gives the impression that these two methodologies are nearly equivalent, whereas the manuscript hints that the results from the two studies may be somewhat different in magnitude (page 6).
The authors compare landmark based and landmark free analyses in the Dp1Tyb mouse model of Down syndrome. The Dp1Tyb mouse model seems to be a near genetic equivalent to the Dp(16)1Yey mouse model that was analyzed by Starbuck et al. (PMC4107150). But, there is very little comparison between the two studies (one sentence in the introduction and one sentence in the results). It would be beneficial to make a comparison in the discussion section between these two studies to relate (verify?) the landmarks and reported changes in Dp1Tyb and Dp(16)1Yey as they compare to normal mice. Of interest may be that the study by Starbuck found differences in the cranial base, whereas the current study found no such differences. Nuances between the two models and experimental methodology between studies should also be emphasized.
The authors state that there is not much known about the development or genetic basis of the Down syndrome craniofacial phenotype. Work has been done in these areas using mouse and fish models of Down syndrome and the authors should acknowledge these advances. See articles from the Reeves laboratory including PMC2820727, PMC2903219, and PMC6027891.
Minor point: two references are missing authors: Med Image Anal 1, 225-243 and Dev Genes Evol 226, 113-137.

Reviewer 3

Advance summary and potential significance to field

Toussaint et al. present and compare a landmark-free morphometrics pipeline used in neuroimaging to an established landmark-based method used commonly on CT data. The authors apply these methods to analyze the, previously undescribed phenotypes of the craniofacial skeleton in Dp1Tyb mutant mice, which serve as a model for Down Syndrome in humans. Similar trends in

© 2021. Published by The Company of Biologists under the terms of the Creative Commons Attribution License (https://creativecommons.org/licenses/by/4.0/).

dysmorphology were observed using either method, where parameters such as computed centroid size and principal component analyses were compared. However, landmark free analysis permitted simpler visualization of change and improved the ability of the researchers to find more discrete changes in areas that traditionally have fewer landmarks, such as the mandible. Further, the higher density control points allowed surface stretch to be used as a way of demonstrating local size changes. Using this method, the authors found deficiencies in the midpalate and auditory bulla that were not captured in landmark-based methods by performing scaled and unscaled surface stretch analyses. Importantly, these data improve accuracy when visualizing relative amplitude in change across the specimen through both static and animated representations. Together, this pipeline overcomes limitations of the landmark-based method such as technical variability and poor coverage in areas with few landmarks while also improving biological insight and reducing the need for expert anatomical understanding.

Comments for the author

This manuscript is appropriate for publication as a Techniques and Resources Article in Development. The resource described is of broad interest to the developmental biology community as it has the potential to be applied to many systems and offers novel biological insight. Significantly, the proposed method improves visualization of morphometric change and is more accessible to non-experts. However, minor comments below should be addressed.

Minor comments:

1. The introduction could be made stronger by addressing the following questions: Why do scale and shape need to be separated in other morphometric methods? How do landmark-free morphometrics avoid separation? Why would developing a new tool that avoids scale and shape separation be advantageous?
While this is touched upon briefly in the results, these points addressed in the introduction would help the reader understand the importance of figure 5.

2. It may be important to introduce the use of centroid size in comparing morphologies and briefly how would be calculated in either method. This would help the reader understand why the difference in absolute magnitude of centroid size between both techniques is significant.

3. One strength of the pipeline presented is the ability to visualize shape differences through animation. Improved visualization techniques are becoming increasingly important when analyzing complex or subtle phenotypes. Exploring the need for such tools in the introduction would further convey the benefits of the system and increase understanding.

4. Fig. 1: While the introduction implies that the arrows generated during atlas construction are momentum vectors, it is not entirely clear whether this is indeed the case in the figure legend.

5. The data not shown addressing overfitting caused by so many measurements should be included in supplementary materials as this is an important control – a point emphasized in the discussion.

6. Male and female specimens are compared in the PCA presented in Fig. 2 E-H however, this is not mentioned in the results. Discussing the sex-based trends recapitulated between both methods could highlight the benefits of using the presented system. While not essential, it would be interesting to identify whether sex impacts the severity of dysmorphology in mutant crania as there are gender differences in patients with trisomy. This insight would add to the biological conclusions offered by this report.

7. Fig. 3: It is not clear in the results whether mean meshes were calculated from samples of both sexes or a single-sex. This should be made clear as, although less important in the crania, PCA in Fig 2.H would suggest that sex is a potential driver of variation in mutant mandibular shape.

8. Supplemental fig. 3: It is not entirely clear in the results or figure legend this displacement map is generated by landmark or landmark-free centroids. This should be added to the text.

© 2021. Published by The Company of Biologists under the terms of the Creative Commons Attribution License (https://creativecommons.org/licenses/by/4.0/).

9. In the discussion, it is written that "The high density of control points was further refined by having them clustered algorithmically at regions of high variability in the samples". This is not reported in the results but is important because this step is a key way in which the landmark-free method can have greater quantitative value. It may, therefore, be important to include in the manuscript body, data addressing whether clustering high-density control points or not, impacts the degree of shape change recorded compared to landmark-based methods.

10. While in the results it is shown that Dp1Tyb mice have smaller auditory bulla.
(AB), it is not discussed how this might relate to hearing and ear phenotypes presented by human trisomy patients. For example, Otis media, present in trisomy patients, is associated with smaller auditory bulla in other craniofacial mouse models e.g. Tcof1. While not essential, discussing this point my strengthen the argument that this allele can be used as a model for understanding craniofacial phenotypes in human and strengthen the biological predictions put forth by this report.

11. In page 11, paragraph 2 it is suggested that the short spatial scale of mapping could make it easier to localize changes in biological processes e.g. proliferation or ECM expansion. While it is reasonable to suggest that the resolution of meshes used in the presented pipeline resolve areas of change relative to the landmark-based method tested, the suggestion that this approach could help predict which morphogenetic mechanism underlies particular dysmorphology is slightly overstated and should be clarified or toned down. This is especially true as W = 2mm.

12. It is not entirely clear from the methods section which step in the github instructions is used to generate the animations. I have to guess that this is what is meant by cyclic deformations of subgroups subheading in the github instructions.
This should be referred to and clarified, if not expanded upon in materials and methods. If there are notes on how to select animation speed, format/organization of animations or labels while generating these files, these should also be included.
Also, can scale bars for the heat map color be included in animations when writing the file?

13. One suggestion in this report is the applicability of this pipeline to other data sets. While not essential, this goal would be better achieved if some discussion was included as to the kind of segmented image data that could be used e.g. cellular morphologies from confocal data? PET data? In situs?

14. Further to 13., the limitations of this pipeline e.g. computing time vs σW, have not been discussed. How computing time compares to landmark-based analysis is also not addressed but is important depending on the resources available to the researcher.

First revision

Author response to reviewers' comments

We thank the reviewers for their many and helpful comments and we have responded to all points as detailed below. We have clarified the text in many places and added a whole new section with figures to better validate our computational pipeline. We have also done a lot of work to improve the useability of the pipeline itself and while this may not show in the manuscript, it will certainly help make this resource more accessible to new users.

We reproduce the reviewers' comments below in full with our detailed responses in green. We are also providing a version of the manuscript itself annotated to indicate where changes have been made in response to reviewers' comments.

Reviewer 1 Advance Summary and Potential Significance to Field:
The method presented potentially provides a significant advance to the field. However, the presentation of the data is sloppy, making evaluation of the method difficult. Additional tests, especially of population variance would improve the evaluation and application of the method.

© 2021. Published by The Company of Biologists under the terms of the Creative Commons Attribution License (https://creativecommons.org/licenses/by/4.0/).

Reviewer 1 Comments for the Author:
The article by Toussaint and colleagues describes new methods for landmark-free morphometric analyses of craniofacial shapes. A landmark-free method for morphometric analysis would be a great addition to the developmental biology community. In this manuscript, some promising data is presented, but further analyses should be included to provide a more thorough comparison of the technique to landmark-based methods, especially one that quantifies variance. Additionally, the figures lack critical information and are presented in inconsistent manners making comparisons and interpretations of the data challenging, if not impossible. This sloppy presentation detracts from a potentially interesting manuscript.

Major points:
1. The first section of the results “Landmark-based and landmark-free . . . “ is not a result. It is a summary of the methods, which they also describe in more detail in the methods. Its inclusion here seems unnecessary and confusing since critical information is lost in the summary. For clarity, I recommend removing this section.
Since this article is intended for the Technique and Resources section of Development we feel that there must be a summary of the methods in the Results section. However, for clarification we have amended the main text (Results, paragraph 2, lines 135-136)

2. In Figure 2, the graphs should be presented on similar scales to facilitate comparison. The PCA plots include different shapes and colors that are not described in the legend, making it impossible to interpret the data.
We have amended the graphs in panels A-D to show normalised centroid size (rather than centroid size) thereby allowing the graphs to be plotted on the same scale, and permitting a direct comparison between the landmark-based and landmark-free methods. We have enlarged the colour key for panels E-H so that it is more conspicuous and also described the colour key in the legend.

3. For the PCA analyses presented in Figure 2, there appear to be differences in population variance in the mandible samples (assuming I properly interpreted the shapes), with the landmark-free sample potentially exhibiting more variance in both WT and mutant mandible populations. As variance is an important measure of any population, any difference in the methods in reporting variance should be noted. Therefore, population variance should be quantified and any difference between the methods should be discussed. An accurate quantification of variance may involve increasing sample size.
We have considered this question carefully and in response have added an entire new section (pp.11-13, lines 262-319 and various points in the Abstract and Discussion) and figures (Fig.6 and Suplementary Fig. S5) to the paper where we analyse a much larger dataset to compare variance measures using the different measures.

4. In Figures 3 and 4 the scale for distance differs by an order of magnitude. In figure 4, the mandible distances go from 0.09 to 1.0 There is also only one scale for landmark-based methods and two for the landmark-free. The color code is also not the same on this distance scales, again making comparisons of the methods difficult.
We thank the reviewer for pointing out the discrepancy in the distance scales. We have now corrected the distance and colour scales so that they are the same for the different figures that use the same method. As we now explain in the text (lines 225-227), the landmark-free method uses a different colour scale because the Deformetrica algorithm does not distinguish between inward and outward deformation, but instead, the directions of deformations are presented by the morphing videos and our stretch mapping figures.

5. The interpretation of the data presented in Figure 5 is that the mutant phenotype is “clearly . . . a growth deficit in the occipital region.” Given that the authors have only investigated one developmental stage and have not assessed proliferation, differentiation, or cell death of any tissues at any time, conclusions about growth cannot be made. Their stretch data may imply some size difference, but without reference to any developmental data, it is a real stretch to say it represents a growth difference. Further, there appear to be quite a number of areas in the skull that show differences in “stretch.” Why is the occipital region singled out? Do the authors have modeling data to show that differential growth in this region of the skull could mimic the shape changes in the DS mice? Overall, this data appears to be over-interpreted and any advantages of this technique are not clearly presented.

© 2021. Published by The Company of Biologists under the terms of the Creative Commons Attribution License (https://creativecommons.org/licenses/by/4.0/).

We have amended the text to clarify that there are changes in three main areas: the occipital region, the facial bones and hard palate (lines 252-256). We have also altered the text to make it clear that by growth we mean size increase, not just cell proliferation, so that it is clear that we do not over-interpret any shape differences as due to specific cellular processes (lines 245-246 and 253).

Minor points:
1. In the Introduction, the authors provide some details on previous work using the DS model mouse phenotypes. They say that Ts65Dn and Dp(16)1 Yey mice were shown to resemble the DS phenotype, and then go on to say that Dp1Yey mice demonstrate statistically significant dysmorphology. What is meant by this? Shouldn't both mouse lines exhibit dysmorphology if they resemble the DS phenotype? Is this the more severe of the two lines? the less severe of the two? I gather the authors are trying to justify their choice to focus on this particular line, but the reasoning is not clear. It seems that the best test of the method would be to use the less severe line. In any case, some rewording of this paragraph would help clarify why the specific model was chosen.
We have re-written this section of the Introduction (lines 86-103) to clarify the genetics of the three strains we refer to (Ts65Dn, Dp(16)1Yey and Dp1Tyb), explaining why the Dp(16)1Yey and Dp1Tyb are better models, and describing the published phenotype in the Dp(16)1Yey mouse strain.

2. The authors should use a consistent terminology for the mice. In the introduction they use both Dp(16)1 Yey and Dp1Yey, while in the discussion they say Dp1Tyb, which I can only assume refers to the same mice.
We have amended the text (lines 96-100 and 109) to correct Dp1Yey to Dp(16)1Yey in all instances and to clarify that the Dp(16)1Yey strain is genetically similar to the Dp1Tyb strain, but the two strains were independently generated by two different groups; we (VLJT & EMCF) made the Dp1Tyb strain which we analyse here.

3. Assuming Dp1Tyb refers to the same mice that they present data for in the paper, they say in the discussion that this mouse model was previously unexamined. Yet in the introduction (as noted in point 1 above), they describe previous work on this mouse.
As clarified above, the Dp1Tyb and Dp(16)1Yey mouse strains mice are different. There have been no previous publications analysing the craniofacial skeleton of Dp1Tyb mice.

4. Some references are listed as numbers
This has been corrected.

5. the inclusion of "tightly localised cranial phenotype" in the title is misleading and also vague. the point of the paper is a method comparison, so it would be more appropriate to have some conclusion about the difference in the methods in the title.
We have amended the title to emphasise the methods-focus of the article

Reviewer 2 Comments for the Author:
The following changes and additions would make this manuscript more suitable for publication:
1. Figure 2 is nearly impossible to read. Additionally, to accurately see the differences between the landmark free and landmark based approaches, the data should be presented on graphs with the same vertical axes.
We have amended the graphs in panels A-D to show normalised centroid size (rather than centroid size) thereby allowing the graphs to be plotted on the same scale, and permitting a more direct comparison between the landmark-based and landmark-free methods and explained normalisation in the text (lines 164-172). We have also clarified the PCA plot key both within the figure and in the legend.

2. As presently constituted, Figure 2 gives the impression that these two methodologies are nearly equivalent, whereas the manuscript hints that the results from the two studies may be somewhat different in magnitude (page 6).
The amended figure highlights the differences between the methods but the main text now clarifies that they are qualitatively very similar.

© 2021. Published by The Company of Biologists under the terms of the Creative Commons Attribution License (https://creativecommons.org/licenses/by/4.0/).

3. The authors compare landmark based and landmark free analyses in the Dp1Tyb mouse model of Down syndrome. The Dp1Tyb mouse model seems to be a near genetic equivalent to the Dp(16)1Yey mouse model that was analyzed by Starbuck et al. (PMC4107150). But, there is very little comparison between the two studies (one sentence in the introduction and one sentence in the results). It would be beneficial to make a comparison in the discussion section between these two studies to relate (verify?) the landmarks and reported changes in Dp1Tyb and Dp(16)1Yey as they compare to normal mice. Of interest may be that the study by Starbuck found differences in the cranial base, whereas the current study found no such differences. Nuances between the two models and experimental methodology between studies should also be emphasized.
We have now added a full paragraph (lines 343-356) comparing the Dp(16)1Yey and Dp1Tyb craniofacial phenotype, pointing out similarities and differences.

4. The authors state that there is not much known about the development or genetic basis of the Down syndrome craniofacial phenotype. Work has been done in these areas using mouse and fish models of Down syndrome and the authors should acknowledge these advances. See articles from the Reeves laboratory including PMC2820727, PMC2903219, and PMC6027891.
We have amended the Introduction to include this background work and to better explain the progression of models (p.4, paragraph 3, lines 87-88).

5. Minor point: two references are missing authors: Med Image Anal 1, 225-243 and Dev Genes Evol 226, 113-137.
This has been corrected.

Reviewer 3 Comments for the Author:
This manuscript is appropriate for publication as a Techniques and Resources Article in Development. The resource described is of broad interest to the developmental biology community as it has the potential to be applied to many systems and offers novel biological insight. Significantly, the proposed method improves visualization of morphometric change and is more accessible to non experts. However, minor comments below should be addressed.

Minor comments:

1. The introduction could be made stronger by addressing the following questions:
Why do scale and shape need to be separated in other morphometric methods?
How do landmark-free morphometrics avoid separation?
Why would developing a new tool that avoids scale and shape separation be advantageous?
While this is touched upon briefly in the results, these points addressed in the introduction would help the reader understand the importance of figure 5.
We have added text to the Introduction (lines 50-53) and Results (lines 240-244) and added a figure (Fig. S4) to signpost the problem of scaling and the need for higher-resolution mapping to better highlight these questions.

2. It may be important to introduce the use of centroid size in comparing morphologies and briefly how would be calculated in either method. This would help the reader understand why the difference in absolute magnitude of centroid size between both techniques is significant.
We have now added text to the section on centroid size (ines 164-172) explaining how it is calculated in the two methods and why there is a difference between the methods.

3. One strength of the pipeline presented is the ability to visualize shape differences through animation. Improved visualization techniques are becoming increasingly important when analyzing complex or subtle phenotypes. Exploring the need for such tools in the introduction would further convey the benefits of the system and increase understanding.
We have amended the Introduction in two places (second and last paragraph) to capture this idea.

4. Fig. 1: While the introduction implies that the arrows generated during atlas construction are momentum vectors, it is not entirely clear whether this is indeed the case in the figure legend.
We have amended the main text (second paragraph of Results) and the Fig.1 legend to clarify that these are indeed momentum vectors.

© 2021. Published by The Company of Biologists under the terms of the Creative Commons Attribution License (https://creativecommons.org/licenses/by/4.0/).

5. The data not shown addressing overfitting caused by so many measurements should be included in supplementary materials as this is an important control – a point emphasized in the discussion.
We have added the overfitting controls as Supplementary Figure S3

6.Male and female specimens are compared in the PCA presented in Fig. 2 E-H, however, this is not mentioned in the results. Discussing the sex-based trends recapitulated between both methods could highlight the benefits of using the presented system. While not essential, it would be interesting to identify whether sex impacts the severity of dysmorphology in mutant crania as there are gender differences in patients with trisomy. This insight would add to the biological conclusions offered by this report.
We have amended the main text (lines 192-195) to point out the similar patterns of sex differences in the PCA plots. Since the effects are only in the mandible and are relatively subtle, we feel that we do not have the statistical power to comment further.

7. Fig. 3: It is not clear in the results whether mean meshes were calculated from samples of both sexes or a single-sex. This should be made clear as, although less important in the crania, PCA in Fig 2.H would suggest that sex is a potential driver of variation in mutant mandibular shape.
We have amended the text (line 192-4) to clarify that the separations were similar in each single sex group and so sexes were pooled for further analysis.

8. Supplemental fig. 3: It is not entirely clear in the results or figure legend this displacement map is generated by landmark or landmark-free centroids. This should be added to the text.
We have amended the legend to Supplementary Fig.S4 to clarify this.

9. In the discussion, it is written that “The high density of control points was further refined by having them clustered algorithmically at regions of high variability in the samples”. This is not reported in the results but is important because this step is a key way in which the landmark-free method can have greater quantitative value. It may, therefore, be important to include in the manuscript body, data addressing whether clustering high-density control points or not, impacts the degree of shape change recorded compared to landmark-based methods.
We agree with Reviewer 3 that the control point optimisation built into the Deformetrica algorithm is an ingenious enhancement. However, since it is not readily user-modifiable we feel that running tests on its effects is beyond the scope of this article .

10. While in the results it is shown that Dp1Tyb mice have smaller auditory bulla. (AB), it is not discussed how this might relate to hearing and ear phenotypes presented by human trisomy patients. For example, Otis media, present in trisomy patients, is associated with smaller auditory bulla in other craniofacial mouse models e.g. Tcof1. While not essential, discussing this point may strengthen the argument that this allele can be used as a model for understanding craniofacial phenotypes in human and strengthen the biological predictions put forth by this report.
We have clarified (lines 253-256) that although the auditory bulla is smaller in Dp1Tyb mice, this reduction in size is in proportion to the overall decrease in skull size. In contrast, there is a disproportionate decrease in size in a region posterior to the auditory bulla. The relationship of the smaller auditory bulla to otitis media remains unclear.

11. In page 11, paragraph 2 it is suggested that the short spatial scale of mapping could make it easier to localize changes in biological processes e.g. proliferation or ECM expansion. While it is reasonable to suggest that the resolution of meshes used in the presented pipeline resolve areas of change relative to the landmark-based method tested, the suggestion that this approach could help predict which morphogenetic mechanism underlies particular dysmorphology is slightly overstated and should be clarified or toned down. This is especially true as σW = 2mm.
We have amended the text (lines 423-425) to clarify and toned down what we mean in this paragraph, explaining that the high-resolution morphometrics is a useful tool for knowing where to look for cell behaviour differences, not a technique for predicting which of those behaviours to look at.

12. It is not entirely clear from the methods section which step in the github instructions is used to generate the animations. I have to guess that this is what is meant by cyclic deformations of

© 2021. Published by The Company of Biologists under the terms of the Creative Commons Attribution License (https://creativecommons.org/licenses/by/4.0/).

subgroups subheading in the github instructions.
This should be referred to and clarified, if not expanded upon in materials and methods. If there are notes on how to select animation speed, format/organization of animations or labels while generating these files, these should also be included. Also, can scale bars for the heat map color be included in animations when writing the file?
We have re-written the relevant section (lines 601-2) to clarify that what we describe is the generation of animations and referred to the specific section of the method as detailed in the Appendix within the Supplementary Materials.

13. One suggestion in this report is the applicability of this pipeline to other data sets. While not essential, this goal would be better achieved if some discussion was included as to the kind of segmented image data that could be used e.g. cellular morphologies from confocal data? PET data? In situs?
We have amended the main text (lines 429-30) to explain that any 2D or 3D dataset with a well-defined contour/surface can be used, including confocal or other types of data.

14. Further to 13., the limitations of this pipeline e.g. computing time vs σW, have not been discussed. How computing time compares to landmark-based analysis is also not addressed but is important depending on the resources available to the researcher.
We have added a sentence in the Methods (504-507) and a paragraph in the Discussion (lines 374-384) to describe the hardware used to do the computations and the computing time taken and describe how the choice of control point number/resolution affects the computation time. In the Discussion we compare the time requirements between the landmark-free and landmark-based analysis

---

Second decision letter

MS ID#: DEVELOP/2020/188631

MS TITLE: A landmark-free morphometrics pipeline for high-resolution phenotyping: application to a mouse model of Down Syndrome

AUTHORS: Nicolas Toussaint, Yushi Redhead, Marta Vidal-Garcia, Lucas Lo Vercio, Wei Liu, Elizabeth Fisher, Benedikt Hallgrimsson, Victor Tybulewicz, Julia Schnabel, and Jeremy Green
ARTICLE TYPE: Techniques and Resources Article

I am happy to tell you that your manuscript has been accepted for publication in Development, pending our standard ethics checks. Please see especially one comment from reviewer#3 requesting addition of a figure callout to help orient the reader.

(On a personal note, please accept my apologies for the delay in getting a decision to you. In part this was due to my own inattention over the holiday, but then was exacerbatedby a technical glitch in the website that had me locked out for the last week!)

Reviewer 1

Advance summary and potential significance to field

The revised version of this manuscript provides a clear and convincing description of a land-mark free method for morphometric analysis. This method has significant potential applications within developmental biology. Importantly, this may lead to an increase in quantitative phenotyping, which is important to understand subtle variations in developmental outcomes.

© 2021. Published by The Company of Biologists under the terms of the Creative Commons Attribution License (https://creativecommons.org/licenses/by/4.0/).

Comments for the author

The authors have provided extensive revisions- the arguments and interpretations are presented clearly and new material has been added to further clarify the value of this method. The figures have been significantly improved- the data is compelling and visually appealing. Overall, the manuscript is suitable for publication as is and will make a significant contribution to the field.

Reviewer 2

Advance summary and potential significance to field

The manuscript by Toussaint et al. "A landmark-free morphometrics pipeline for high-resolution phenotyping: application to a mouse model of Down Syndrome" details a new landmark-free approach to quantifying differences in craniofacial morphometry. The details of the technique, procedure, and results are well documented. The authors compared the current methodology with those used previously in the field and demonstrated significant improvement over the status quo. The data have been compared and contrasted to similar analyses. This paper will be a seminal study to which others in the field will refer.

Comments for the author

The authors have addressed my concerns from the previous review.

Reviewer 3

Advance summary and potential significance to field

Morphometrics in developmental biology and medicine serve as important diagnostic tools, of which many rely on defined landmarks. In structures with few features, insufficient landmarks decrease the ability to quantify shape change across specimens. Toussaint et al. present a landmark free morphometrics pipeline that has both operational and computational advantages over some previous methods. Further, the reported pipeline is used to characterise cranial skeletal shape change in a mouse model of Down Syndrome demonstrating phenotypic changes that are reflected in human patients. Further, analysis of Diversity Outbred mice suggest that allometry and sexual dimorphism may underlie some aspects of cranial skeleton variation across a population.

Comments for the author

This reviewer believes reviewers comments have been addressed sufficiently to warrant publication.

1 minor comment: add a call out in the final paragraph of the results to Fig. 6D to help the reader.

Figures are significantly better.

© 2021. Published by The Company of Biologists under the terms of the Creative Commons Attribution License (https://creativecommons.org/licenses/by/4.0/).
